# COXFA4L2 upregulation preserves residual cytochrome *c* oxidase activity in *COXFA4*-related Leigh-like encephalopathy

**DOI:** 10.1038/s41467-026-73455-9

**Published:** 2026-05-30

**Authors:** Micol Falabella, Sandra Lopez Calcerrada, Jana Aref, Jiaze Gao, William L. Macken, Chiara Pizzamiglio, Renata Kabiljo, Anna Lucia Francavilla, Pauline Gaignard, Antoine Pouzet, Jonathan Levy, Giulia Barcia, Jamie K. Leighton, Efstathia Chronopoulou, Germaine Pierre, Riza Köksal Özgül, Ali Dursun, Rebecca Halligan, Helen Mundy, Javeria Raza Alvi, Tipu Sultan, William James Craigen, Lisa Emrick, Jill A. Rosenfeld, Gehad Elmakkawy, JiHye Kim, Joseph J. Gleeson, Aboulfazl Rad, Gabriela Oprea, Maqbool Hussain, Khalil Ur Rehman, Sadia Riaz, Robert W. Taylor, Vincent Procaccio, Maha S. Zaki, Erika Fernandez-Vizarra, Ciro Leonardo Pierri, Michael G. Hanna, Henry Houlden, Reza Maroofian, Cristina Ugalde, Jan-Willem Taanman, Robert D. S. Pitceathly

**Affiliations:** 1 Department of Neuromuscular Diseases, University College London Queen Square Institute of Neurology, London, UK; 2 Instituto de Investigación Hospital 12 de Octubre, Madrid, Spain; 3 NHS Highly Specialised Service for Rare Mitochondrial Disorders, Queen Square Centre for Neuromuscular Diseases, The National Hospital for Neurology and Neurosurgery, London, UK; 4 Laboratory of Biochemistry, Structural and Molecular Biology, Department of Pharmacy – Pharmaceutical Sciences, University of Bari “Aldo Moro”, Via E, Orabona 4, Bari, Italy; 5 Service de Biochimie, CHU Bicêtre, AP-HP, Université Paris-Saclay, Centre de Référence des Maladies Mitochondriales, Filière Filnemus, Le Kremlin-Bicêtre, France; 6 Laboratoire de Biologie Médicale Multisite SeqOIA-FMG2025, Paris, France; 7 UMR-S 1180, CARPAT, Inserm/Université Paris-Saclay, Orsay, France; 8 Department of Genetics, APHP-Robert Debré University Hospital, Paris, France; 9 Service de Médecine Génomique des Maladies Rares, APHP Centre, Hôpital Necker-Enfants Malades, Paris, France; 10 Université Paris Cité, Imagine Institute, INSERM, Paris, France; 11 Mitochondrial Research Group, Translational and Clinical Research Institute, Faculty of Medical Sciences, Newcastle University, Newcastle upon Tyne, UK; 12 Department of Inherited Metabolic Disease, Division of Women’s and Children’s Services, University Hospitals Bristol and Weston NHS Foundation Trust, Bristol, UK; 13 Institute of Child Health, Department of Pediatric Metabolism, Faculty of Medicine, Hacettepe University, Ankara, Türkiye; 14 Department of Paediatric Inherited Metabolic Diseases, Evelina London Children’s Hospital, Guy’s and St Thomas’ NHS Foundation Trust, London, UK; 15 Department of Pediatric Neurology, Institute of Child Health, Children Hospital Lahore, Lahore, Pakistan; 16 Department of Molecular and Human Genetics, Baylor College of Medicine, Houston, TX USA; 17 Baylor Genetics, Houston, TX USA; 18 Human Genetics Department, Medical Research Institute, Alexandria University, Alexandria, Egypt; 19 3billion Inc., Seoul, South Korea; 20 Rady Children’s Institute for Genomic Medicine, San Diego, CA USA; 21 Department of Neurosciences and Pediatrics, University of California, San Diego, San Diego, CA USA; 22 Arcensus Diagnostics, Rostock, Germany; 23 Cellular and Molecular Research Center, Sabzevar University of Medical Sciences, Sabzevar, Iran; 24 FCPS Paediatrics, Children Hospital PIMS, Islamabad, Pakistan; 25 Town Women and Children Hospital, Peshawar, Pakistan; 26 PCPS Paediatrics and Neonatology, Children Hospital PIMS Islamabad, Islamabad, Pakistan; 27 NHS Highly Specialised Service for Rare Mitochondrial Disorders of Adults and Children, Newcastle upon Tyne Hospitals NHS Foundation Trust, Newcastle upon Tyne, UK; 28 University of Angers, MitoLab, Unité MITOVASC, UMR CNRS 6015, INSERM U1083, SFR ICAT, University Hospital of Angers, Angers, France; Department of Genetics, University Hospital of Angers, Angers, France; 29 Clinical Genetics Department, Human Genetics and Genome Research Institute, National Research Centre, Cairo, Egypt; 30 Department of Biochemistry and Molecular and Cellular Biology, Faculty of Health and Sport Sciences, University of Zaragoza, Huesca, Spain; 31 Centro de Investigación Biomédica en Red de Enfermedades Raras (CIBERER), Madrid, Spain; 32 Centro de Investigaciones Biológicas Margarita Salas (CIB-CSIC), Madrid, Spain; 33 Department of Clinical and Movement Neurosciences, University College London Queen Square Institute of Neurology, London, UK

**Keywords:** Energy metabolism, Neuromuscular disease, Mechanisms of disease

## Abstract

Primary mitochondrial diseases (PMDs) affect approximately 1 in 4300 individuals and cause early-onset neuromuscular and multisystem dysfunction with reduced lifespan. They result from pathogenic variants in mitochondrial or nuclear DNA that impair oxidative phosphorylation. Cytochrome *c* oxidase (COX; complex IV) deficiency is a well-established cause of PMD, leading to a broad spectrum of phenotypes. COXFA4 (cytochrome *c* oxidase subunit FA4), formerly NDUFA4, is a nuclear-encoded COX subunit, but its role in disease remains poorly defined. We report the largest genetically confirmed cohort of COXFA4-related PMD to date, comprising 13 individuals from 12 families with biallelic pathogenic *COXFA4* variants. All present with Leigh-like encephalopathy and complete loss of COXFA4 protein; however, patient-derived fibroblasts retain residual COX activity, with upregulation of COXFA4L2 (cytochrome *c* oxidase subunit FA4-like 2), a poorly characterised paralog. Here, we show that COXFA4 is a late-stage COX assembly subunit and identify a paralog-mediated compensatory mechanism with translational potential.

## Abstract (web summary)

Falabella et al. report the largest cohort of individuals with biallelic *COXFA4* variants associated with Leigh-like encephalopathy and show that the paralog COXFA4L2 partially compensates for complex IV deficiency, suggesting a potential therapeutic strategy.

## Introduction

Mitochondrial oxidative phosphorylation (OXPHOS) is essential for cellular energy production, particularly in high-energy demanding tissues such as the brain, which consumes approximately 20% of the total body energy^1^. In neurons, ATP generation primarily depends on OXPHOS^2^, highlighting the key role of mitochondrial function in brain physiology and homeostasis. Cytochrome *c* oxidase (COX; complex IV) is localised in the inner mitochondrial membrane where it catalyses the transfer of electrons from cytochrome *c* to molecular oxygen, generating the proton gradient that drives ATP synthesis^3^. Human COX consists of 14 subunits encoded both in the mitochondrial DNA (mtDNA) and the nuclear DNA (nDNA)^4^. The three mtDNA-encoded subunits form the catalytic core, while the remaining 11 nDNA-encoded subunits contribute to structural integrity and regulation of the holoenzyme^5^. COX biogenesis is a complex, multistep process in which numerous nuclear-encoded assembly factors are involved, coordinating subunit stabilisation, membrane integration, insertion of metal catalytic centres and assembly^6^. Importantly, seven nuclear-encoded COX subunits are expressed as tissue-specific or conditionally regulated isoforms^7,8^. These isoforms are hypothesised to regulate COX activity, facilitating metabolic adaptation to the diverse energy demands and physiological conditions of different tissues^5,9^.

Pathogenic variants in genes encoding COX subunits, their isoforms or assembly factors are well-established causes of primary mitochondrial disease (PMD)^10^. These variants typically result in isolated COX deficiency by disrupting COX biogenesis, maturation, structural stability, and/or enzymatic function, leading to impaired OXPHOS. Clinical phenotypes are heterogeneous, and include myopathy, cardiomyopathy, hepatic encephalopathy, and Leigh-like syndrome, with onset and severity ranging from neonatal lethality to milder, adult-onset manifestations^10,11^. Variants in COX assembly factors are the most frequent genetic causes of COX deficiency^12^. Pathogenic variants in mtDNA-encoded subunits are less common, and those affecting nuclear-encoded structural subunits are even rarer^10,13^. This highlights the essential role of these subunits for COX assembly, structural stability, and enzymatic function and suggests that most variants in the structural components of COX are incompatible with life^14^. To date, only a limited number of pathogenic variants have been reported in nuclear-encoded subunits^15–22^, including *COXFA4* (cytochrome *c* oxidase subunit FA4)^10^.

COXFA4, formerly known as NDUFA4, is the most recently described structural subunit of COX. Although initially misclassified as a subunit of NADH:ubiquinone oxidoreductase (complex I), data obtained from patient-derived tissues, immortalised cell lines, and high-resolution cryo-electron microscopy have unequivocally established COXFA4 as a bona fide COX subunit^4,23–25^. COXFA4 has been proposed to participate in the late assembly stages of the ‘free’ COX holoenzyme^26,27^ and localises peripherally, which potentially explains its dissociation from COX under specific detergent conditions during sample preparation^23^. COXFA4 has two paralogs: COXFA4L2 (cytochrome *c* oxidase hypoxia associated subunit FA4L2) and COXFA4L3 (cytochrome *c* oxidase associated subunit FA4L3; formerly C15orf48; also known as MOCCI). COXFA4L3 is upregulated during spermatogenesis and in response to inflammatory stimuli, displacing COXFA4 from the COX complex and promoting its degradation^28–31^. In contrast, COXFA4L2 is transcriptionally induced under hypoxic conditions^32^; however, its role in PMDs remains poorly understood. Biallelic pathogenic variants in *COXFA4* (RefSeq: NM_002489.4) cause isolated COX deficiency, leading to a diverse range of clinical phenotypes. These include neurodevelopmental delay, lactic acidosis, and Leigh-like encephalopathy^23,33^. Remarkably, despite the complete loss of COXFA4 protein in patient-derived skeletal muscle and skin fibroblasts, partial COX activity is retained, suggesting adaptive, previously uncharacterised compensatory mechanisms.

Here, we report the largest cohort of COX-deficient PMD cases caused by a nuclear-encoded subunit of complex IV, comprising 13 affected individuals from 12 unrelated families harbouring novel biallelic *COXFA4* variants. All individuals presented with Leigh-like encephalopathy associated with complete loss of COXFA4 protein expression. Using an integrated approach that combined bioenergetic analyses of patient-derived fibroblasts, transcriptomic profiling, and kinetic studies of COX assembly, we identified a previously unrecognised compensatory mechanism involving the upregulation of COXFA4L2. Additionally, our data provide insights into the late-stage role of COXFA4 in COX holoenzyme, with secondary consequences for respiratory supercomplexes (SCs) abundance. These findings provide insights into the pathophysiology of *COXFA4*-associated PMD, significantly expand the spectrum of pathogenic *COXFA4* variants, and reveal a mitochondrial adaptive response involving upregulation of COXFA4L2 to help sustain cellular energy production.

## Results

### Identification of candidate pathogenic *COXFA4* variants

**Fig. 1:**
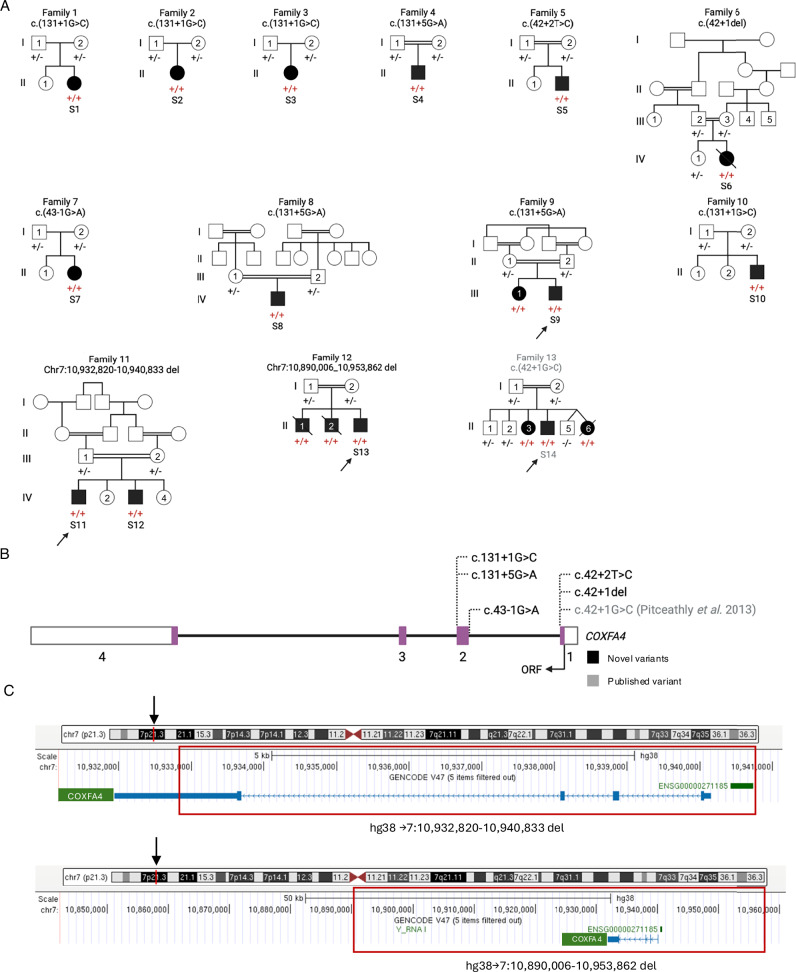
Family pedigrees and details of *COXFA*4 variants. **A** Genetic pedigrees of 13 unrelated families with homozygous *COXFA4* variants. Affected individuals (S1–S14) are shown by filled symbols; circles represent females, and squares represent males. Double horizontal lines indicate consanguinity, and probands are marked with arrows. Subject S14 (Family 13), previously reported, is shown in grey. **B** Schematic representation of the *COXFA4* gene, highlighting the location of the identified variants. Variants reported in this study are represented in black, while the previously reported variant is in grey. **C** Modified snapshots from the University of California, Santa Cruz (UCSC) Genome Browser (GRCh38/hg38) highlighting two deletions within the boxed regions: chr7:10,932,820–10,940,833 (top panel, identified in S11 and S12) and chr7:10,890,006–10,953,862 (bottom panel, identified in S13). Arrows indicate the chromosome region containing the *COXFA4* gene.

Through the GeneMatcher platform, the RD-Connect Genome-Phenome Analysis Platform (GPAP), the 100,000 Genomes Project, the NHS England Genomic Medicine Service and collaboration with national and international clinical and research groups, we identified 13 affected individuals from 12 unrelated families with biallelic variants in *COXFA4* (Fig. 1A; and Tables 1 and 2). All individuals presented with a Leigh-like neurodevelopmental disorder, characterised by progressive developmental delays, neurodegeneration, muscle weakness, and lactic acidosis. This cohort included Subject 14 (S14), which was previously reported^23^; however, additional functional analyses performed in this study provided further insight into the molecular and phenotypic consequences of COXFA4 deficiency in this case. Overall, we describe five novel splice site variants identified in 10 independent families (Fig. 1A, B) and two novel homozygous deletions in the *COXFA4* gene. The first deletion spanned 8013 bp and partially overlapped the gene, whereas the second was a larger deletion of 63,856 bp on chromosome 7, which completely overlapped the *COXFA4* locus (Fig. 1C). None of the variants reported were present in a homozygous state in gnomAD v.4.1.0. *COXFA4* variants were confirmed using Sanger sequencing (Supplementary Fig. 1A). The c.131+1 G > C variant was identified in four unrelated families of Sri Lankan origin (Families 1, 2, 3, and 10; Subjects S1, S2, S3, and S10). Similarly, c.131+5 G > A was found in three independent families of Afghan origin (Family 4; S4), Pakistani (Family 8; S8), and Pathan (Family 9; S9). The recurrence of these *COXFA4* variants suggests a possible founder effect and genetic relatedness. To investigate this further, we performed a DNA microarray analysis on cases where DNA was available. The haplotype analysis showed that S1 and S2 shared a haplotype, suggesting a common ancestry, likely more distant than a second cousin relationship (Supplementary Fig. 1B). Similarly, S4 and S8 also demonstrated overlapping segments, supporting the possibility of genetic relatedness (Supplementary Fig. 1C). Consanguinity was reported in 7 of 12 families (54%), while the remaining five families were non-consanguineous.

**Table 1:** Clinical, radiological, biochemical, and molecular features of the six subjects with biallelic *COXFA4* variants whose primary fibroblasts were used for functional analysis

Subject (Family)	S1 (F1)	S2 (F2)	S3 (F3)	S4 (F4)	S5 (F5)	S14 (F13)
Variant	c.131+1 G > C	c.131+1 G > C	c.131+1 G > C	c.131+5 G > A	c.42+2 T > C	c.42+1 G > C
Sex/Age (y)	Female/29	Female/3	Female/3	Male/9	Male/17	Male/45
Ethnicity	Sri Lankan	Sri Lankan	Sri Lankan	Afghan	Turkish	Pakistan
Consanguinity	N	N	N	Y	Y	Y
Onset	Birth	Birth	Birth	Birth	Birth	Birth
Initial symptom	Hypotonia, poor feeding, lethargic	Respiratory distress (birth) raised lactate	Respiratory distress (birth)	Born at 32/40, respiratory distress, NGT fed	Respiratory distress (birth) feeding difficulties	Lactic acidosis (birth), spastic diplegia (2 y)
Progression	Moderate	Slow	Slow	Slow	Moderate	Slow
Neuromuscular	Proximal muscle weakness, non-ambulatory	Hypotonia	Muscle weakness, ptosis	Exercise intolerance, limb contractures	Myopathic face, non-ambulatory	Sensory axonal neuropathy, dysphagia
Respiratory	OSA with nocturnal CPAP	–	–	–	–	–
Cardiology	WPW pattern, mild LV hypertrophy	Biventricular hypertrophy	Persistent patent ductus arteriosus	–	Concentric LV hypertrophy	–
Endocrinology	Diabetes	–	Growth retardation	–	Low stature	Primary hyperparathyroidism, low stature
Gastrointestinal	–	Vomiting, feeding difficulties	–	Feeding difficulties at birth (now improved)	Feeding difficulties	Constipation
Ophthalmology	Nystagmus	Cataract	–	Nystagmus	–	–
Neurology	Spasticity, cerebellar ataxia, dysarthria, limb dystonia, mild head and arm tremor	–	Ataxia	LL spasticity, cerebellar ataxia, dysarthria, GTC seizures (2 yrs 10 m)	LL spasticity, clonus, brisk reflexes, extensor plantar responses	LL spasticity, brisk reflexes, extensor plantar responses, GTC/myoclonic seizures (13 yrs)
ID	Moderate	N	N	Severe	Severe	Moderate
Gross motor	Sitting 1 y 6 m, walking 2 y, lost at 13 y	Cruising furniture 1 y 1 m	Sitting 7 m, walking 2 y	Sitting 5 m, walking 1 y 2 m	Head supported 4 m, sitting 2 y	–
Speech	Delayed	15 words (1 y 1 m)	13 m	Delayed	1 word (2 y)	Delayed
DevR	Y	–	–	Y	–	–
FBD/SCA	–	–	RVF, prenatal pyelocaliceal dilatation	–	High-arched palate	–
Muscle biopsy	↓ CIV	N/A	N/A	↓ CIV	N/A	↓ CIV
Brain MRI	Thin CC, cerebral atrophy, WM involvement (25 yrs)	Bilateral small lesions in the posterior parietal periventricular WM (6 d)	Mild cerebral atrophy, bilateral, symmetrical signal abnormalities in the supratentorial and subtentorial WM, putamen, brainstem (24 m)	Multifocal symmetrical WM signal abnormalityinvolving the central WM of the CC andboth dentate nuclei (16 m)	N/A	Normal (17 yrs), T2 hyperintensities in the deep WM (25 yrs)
Venous lactate	↑	↑	↑	↑	↑	↑

**Table 2:** Clinical, radiological, biochemical, and molecular features of the eight subjects with biallelic *COXFA4* variants whose primary fibroblasts were not available for analysis

Subject (Family)	S6 (F6)	S7 (F7)	S8 (F8)	S9 (F9)	S10 (F10)	S11 (F11)	S12 (F11)	S13 (F12)
Variant	c.42+1del	c.43-1 G > A	c.131+5 G > A	c.131+5 G > A	c.131+1 G > C	chr7:10932820 – 10940833del	chr7:10932820 – 10940833del	chr7:10890006-10953862del
Sex/Age (y)	Female/4	Female/17	Male/10	Male/8	Male/9	Male/17	Male/12	Male/2
Ethnicity	North African	Hispanic	South Asian	Asian	Sri Lankan	North African	North African	South Asian
Consanguinity	Y	N	Y	Y	N	Y	Y	Y
Onset	Birth	Birth	Birth	Birth	Infancy	Birth	Infancy	Birth
Initial symptom	Hypotonia, PF, FTT	Hypotonia, meconium aspiration, RDS, PH, WPW, SVT, nystagmus	--	RDS, pneumonia	GDD, FTT	RDS	GDD, FTT	--
Progression	Moderate	Slow	Slow	Moderate	Slow	Moderate	Moderate	Severe
Neuromuscular	MW, hypotonia, non-ambulatory	MW, hypotonia	--	Mild scoliosis, LC (ankle)	MW, hypotonia, LC, EI (7 y)	Mild MA	Mild MA	--
Respiratory	RCI	RCI, asthma	--	--	--	--	--	--
Cardiology	--	SVT, WPW, closed PDA	--	--	--	--	--	--
Endocrinology	--	GH deficiency, osteopenia	--	--	--	--	--	--
Gastrointestinal	Constipation	Constipation	--	--	Vomiting, PEG-fed	Constipation	--	--
Ophthalmology	--	Mild OA, nystagmus	--	Convergent L eye squint	--	--	--	--
Neurology	--	Ataxia, dysarthria, progressive gait abnormality	--	Spasticity in LL, pyramidal signs (brisk reflexes, plantar responses extensor), dystonia of lower limb	Spasticity in LL, pyramidal signs (plantar responses extensor), oral dyskinesia, dystonia	Spasticity in LL, pyramidal signs (brisk reflexes, plantar responses extensor), gait ataxia, dysarthria, dystonia	Mild spasticity in LL, pyramidal signs (brisk reflexes, plantar responses extensor), dystonia	--
ID	Severe	Moderate	Y	Mild	Severe	Severe (ADHD, autism)	Moderate (ADHD)	Y
Gross motor	Sitting (11 m)	Sitting 12 m, walking 5 y	Delayed	Sitting 8 m, walking 13 m, regressed 6 y	Sitting 2 y, no walking	Sitting 2 y, walking 5 y	Sitting 1.5 y, walking 3 y	Delayed
Speech	No speech	1^st^ word (2 y), speaks in short sentences	Delayed	No speech	1 word (1.5 y), sentence (8.5 y)	1 word (6 y), 10 words (17 y)	1 word (4 y), sentence (12 y)	Delayed
DevR	Y	--	Y	Y	--	--	--	--
FBD/SCA	Mild facial dysmorphism	--	Microcephaly, long philtrum	--	--	Small head, narrow FH, down slanting palpebral fissures, thick EB, prominent nose, short philtrum, thin/everted lips, broad teeth, retruded hypoplastic mandible, large, protruding ears	High FH, thick EB, prominent nose, short philtrum, thick lips, straight upper lip and everted lower lip, retruded hypoplastic mandible, protruding ears	--
Muscle biopsy	N/A	↓ CI, ↓CIII and CIV	N/A	N/A	↓ CIV	N/A	N/A	N/A
Brain MRI	T2/FLAIR signal changes with restricted diffusion: periventricular WM, CC, cerebellar dentate nuclei, middle cerebellar peduncles (2 y)	Post supratentorial WM volume loss with thinning + mineralisation of the posterior CC, patchy T2 hyperintensities, cystic encephalomalacia, L hippocampal atrophy with T2 hyperintensity. Ventricular enlargement (14 y), new T2 hyperintensity pons (16 y)	T2/FLAIR high signals in periventricular WM, brainstem, cerebellum, and anterior half of CC. Periventricular cystic changes (2 y)	Bilateral symmetrical T2 high signal in bilateral periventricular, anterior half of CC, medulla oblongata, and cerebellum with few cystic periventricular areas	Normal (6 m)	Normal	Normal	--
Venous lactate	↑	↑	--	--	↑	↑	↑	--

Abbreviations: *ADHD* Attention deficit hyperactivity disorder, *CC* Corpus callosum, *CIV* Cytochrome c oxidase, *DevR* Developmental regression, *EB* Eyebrows, *EI* Exercise intolerance, *F* Family, *FBD/SCA* Facial/body dysmorphisms/structural congenital abnormalities, *FH* Forehead, *FTT* Failure to thrive, *GDD* Global developmental delay, *GH* Growth hormone, ID = Intellectual disability, *L* left, *LC* Limb contracture, *LL* Lower limbs, *m* months, *MA* Muscle atrophy, *MW* Muscle weakness, *N* No, *N/A* Not available, *OA* Optic atrophy, *PDA* Patent ductus arteriosus, *PEG* Percutaneous endoscopic gastrostomy, *PF* Poor feeding, *PH* Pulmonary hypertension, *Post* posterior *RCI* Recurrent chest infections, *RDS* Respiratory distress syndrome, *S* Subject, *SVT* Supraventricular tachycardia, *WM* White matter, *WPW* Wolff-Parkinson-White syndrome; *Y* Yes, *y* years.

### Clinical presentations of individuals with biallelic *COXFA4* variants

**Fig. 2:**
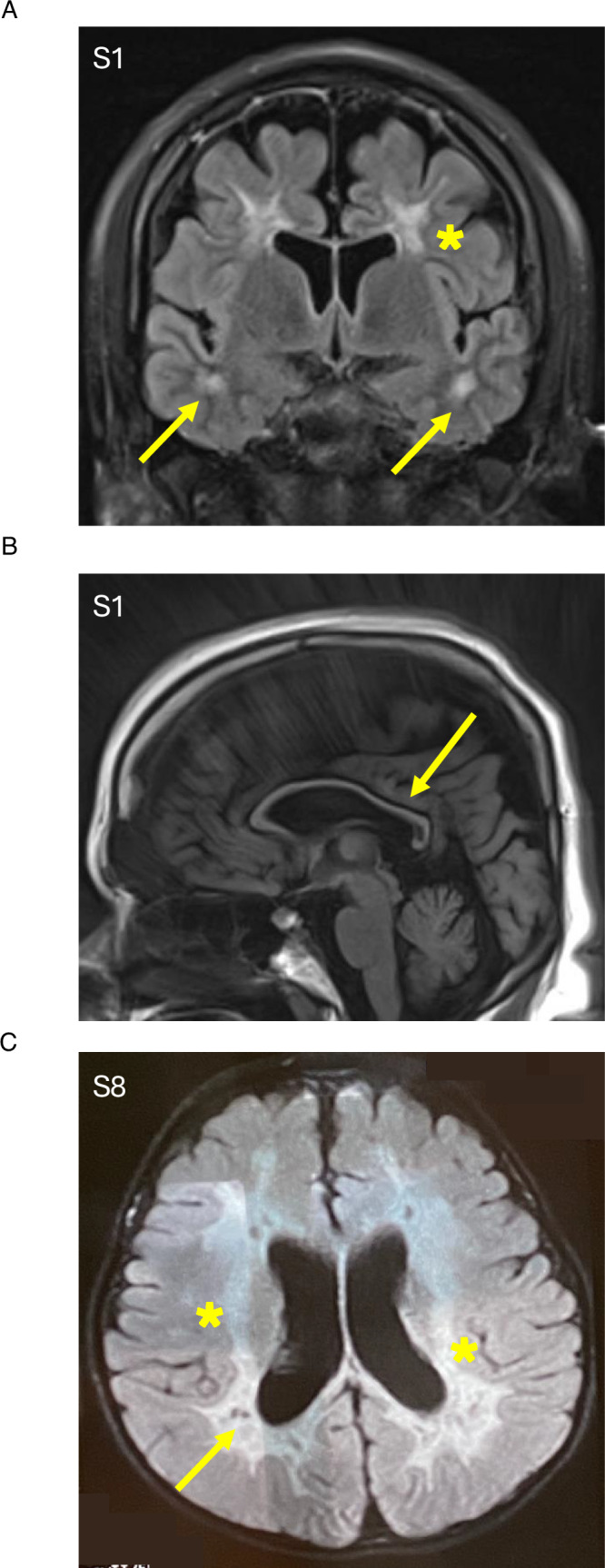
Representative brain MRI findings in *COXFA4* affected subjects. **A**,** B** Subject S1 (Family 1), age 25 years. **A** Coronal FLAIR sequence demonstrating marked periventricular white matter hyperintensities (asterisk) extending into the temporal white matter (arrows). **B** Sagittal view highlighting corpus callosum atrophy. **C** Subject S8 (Family 8), age 2 years. Axial FLAIR sequence showing bilateral, symmetrical hyperintensities in periventricular white matter (asterisk). A few small periventricular cystic changes were also visible (arrows).

All 13 individuals with biallelic *COXFA4* variants presented with Leigh syndrome spectrum disorder. Common clinical features included neonatal or early infantile hypotonia, progressive motor impairment, spasticity, global developmental delay, and varying degrees of intellectual disability. Additional neurological manifestations included cerebellar ataxia (S1, S3, S4, S7, S11), dystonia (S1, S9, S11, S12), ptosis (S3), nystagmus (S4, S7), dysarthria (S1, S7, S11), pyramidal signs (S9–S12), peripheral neuropathy (S14), developmental regression (S1, S9), and seizures (S4, S14). Representative brain magnetic resonance imaging (MRI), images available for a subset of individuals (S1 and S8), revealed symmetrical white matter abnormalities, predominantly affecting the periventricular, corpus callosum, brainstem, and dentate regions (Fig. 2). Systemic involvement was also observed, including cardiomyopathy or conduction abnormalities (S1-3, S5, S7), bulbar dysfunction (S5, S14), skeletal abnormalities such as scoliosis (S5, S9), and endocrine or metabolic features (S1, S7, S14). Respiratory chain enzyme analyses of muscle tissues (S1, S4, S7, S10 and S14) showed a profound reduction in COX activity. Detailed clinical descriptions for individuals whose fibroblasts were available for further functional characterisation are included in Supplementary Information and Table 1. The remaining clinical summaries are presented in Table 2.

### *COXFA4* splice site variants lead to aberrant splicing

In 11 of the 13 families (85%), we identified splice-site variants predicted to alter mRNA splicing, based on the SpliceAI prediction algorithm. To functionally validate these predictions, we performed RNA sequencing (RNA-seq) in cases where fibroblast-derived RNA was available (S1-5 and S14, Supplementary Figs. 2–4).

The c.131+1 G > C variant is located at the donor site of intron 2. SpliceAI predicted loss of this canonical splice donor site (donor loss delta score = 0.98) and loss of the upstream intron 1 acceptor site (acceptor loss delta score = 0.97), with no activation of alternative splice sites. This was predicted to result in the complete loss of canonical splicing of exon 2, producing aberrant transcripts and impairing COXFA4 function. Similarly, the homozygous intronic variant c.131+5 G > A, although outside the canonical splice donor site, was predicted to impair splicing, with donor and acceptor loss delta scores of 0.65 and 0.66, respectively. Again, no alternative splice sites were predicted, consistent with the loss of canonical splicing and exon 2 skipping. RNA-seq analysis supported these in silico predictions. In S1 and S2 (c.131+1 G > C), exon 2 was absent, indicating complete exon skipping compared to controls (Supplementary Figs. 2A, B and 3A). In S3 (c.131+1 G > C), exon 2 was included in 353/2556 reads (13.8%), indicating a minor wild-type leakage (Supplementary Fig. 3A). In S4 (c.131+5 G > A), exon 2 was detected in 85/2350 reads (3.6%), again suggesting predominant exon skipping with low-level transcript leakage (Supplementary Figs. 2A and B).

Both homozygous splice-site variants, c.42+2 T > C and c.42+1 G > C, were predicted to abolish the canonical donor site of exon 1, with high donor loss delta scores (0.98 and 0.99, respectively). In both cases, the predicted loss of an acceptor site was minimal. However, SpliceAI identified a cryptic donor site would be activated just downstream from the native donor (c.42+2 T > C; donor gain delta score = 0.87 2 bp down stream,c.42+1 G > C; donor gain delta score = 0.83 three bp downstream. Activation of these cryptic sites is predicted to disrupt the reading frame and compromise the integrity of the *COXFA4* transcript. Two additional donor-gain predictions were also detected, though with lower confidence. RNA-seq analysis of S5 (c.42+2 T > C) and S14 (c.42+1 G > C) confirmed the use of an alternative donor site, resulting in retention of 4 bp from intron 1 before splicing to the canonical acceptor of exon 2. This aberrant transcript represented the majority of reads (93.6%, 1397/1493 reads in S5 and 62.5%, 843/1348 reads in S14; Supplementary Fig. 4A), consistent with partial intron retention. In addition, a second cryptic donor site was detected at a lower frequency in both individuals. This secondary cryptic donor site at 10,939,508 (hg38) was also supported by 40 reads, resulting in inclusion of 508 bp of intron 1 (10,939,508 − 10,940,016) before splicing to exon 2 (Supplementary Fig. 4B).

### Loss of COXFA4 induces an upregulation of the paralog

#### COXFA4L2

**Fig. 3:**
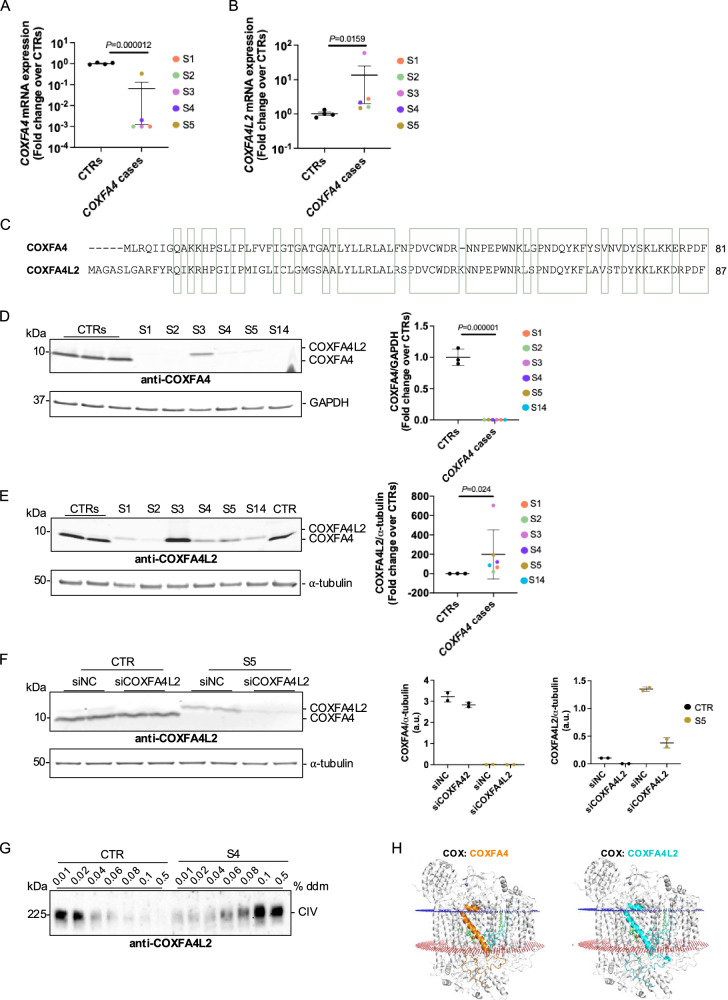
COXFA4 and COXFA4L2 expression in *COXFA4* patient-derived primary fibroblasts. **A** Quantitative real-time PCR (qPCR) analysis of *COXFA4* mRNA, presented on a log_10_ scale and normalised to the housekeeping gene *B2M*. Each data point represents an independent biological sample (CTRs *n* = 4; patients *n* = 5). Statistical significance was assessed using an unpaired two-tailed Student’s t-test. **B** qPCR analysis of *COXFA4L2* mRNA showing upregulation in patient fibroblasts. Data are presented on a log_10_ scale and normalised to the housekeeping gene *B2M*. Each data point represents an independent biological sample (CTRs *n* = 4; patients *n* = 5). Statistical significance was assessed using an exact two-tailed Mann–Whitney test. **C** Sequence alignment of human COXFA4 and COXFA4L2 proteins, highlighting identical residues. **D** Immunoblot analysis of fibroblast lysates from three healthy controls (CTRs) and six COXFA4 patients (S1–S5 and S14). Membranes were probed with a COXFA4 antibody recognising both COXFA4 and COXFA4L2 (Stratech Scientific, C16821-ABT). The absence of COXFA4 is observed in all patient samples, with a higher molecular weight band corresponding to COXFA4L2, particularly in patient S3. GAPDH was used as a loading control. **E** Immunoblot analysis of COXFA4L2 expression in the same samples as panel (**D**) probed with a COXFA4L2 antibody (Proteintech, 16480-1-AP), which also detects COXFA4. A band of higher molecular weight than COXFA4 is detected in all patient samples. α-tubulin was used as a loading control. **F** Immunoblot analysis of COXFA4 and COXFA4L2 expression in primary fibroblasts from one healthy control (CTR) and one COXFA4 patient (S5), following transient knockdown of COXFA4L2 (siCOXFA4L2) or non-targeting negative control siRNA (siNC). Selective reduction of the upper band after COXFA4L2 knockdown confirms its identity as COXFA4L2. α-tubulin was used as a loading control. **G** Blue-native PAGE analysis following n-dodecyl β-D-maltoside (DDM) titration to assess the detergent-dependent stability of COXFA4- and COXFA4L2-containing COX. Protein from primary fibroblasts of healthy control (CTR) and patient S4 were extracted with varying concentrations of DDM, resolved on 8-16% gradient blue-native gels, and probed for COXFA4L2. **H** 3D models of the cytochrome *c* oxidase (COX, white) containing either COXFA4 (left, orange) or COXFA4L2 (right, cyan). Protein-bound cofactors and associated lipids are shown as colored sticks and spheres. The blue and red discs represent the outer and inner leaflets of the inner mitochondrial membrane (IMM), facing the intermembrane space (IMS) and mitochondrial matrix, respectively. Error bars indicate standard deviation (SD), and data are presented as mean ± SD. Each data point represents an independent biological sample, with exact sample sizes and statistical tests indicated in the relevant panels.

*COXFA4* is a highly conserved gene with two known paralogs, *COXFA4L2* and *COXFA4L3*. To assess the presence of a potential compensatory mechanism in response to *COXFA4* loss, we quantified the mRNA expression levels of *COXFA4*, *COXFA4L2*, and *COXFA4L3* by qPCR in patient-derived fibroblasts (Fig. 3). *COXFA4* full-length transcripts were undetected in all patient samples, except for S5, where residual mRNA was observed (Fig. 3A). This residual mRNA in S5 likely reflects the presence of a cryptic donor site, resulting in an aberrant transcript detected in 93.6% of RNA-seq reads (Supplementary Fig. 4A). Interestingly, *COXFA4L2* mRNA expression was significantly upregulated in all cases, particularly in S3 (Fig. 3B), suggesting a potential compensatory response to *COXFA4* loss. *COXFA4L3* transcripts were not detected in controls and *COXFA4*-deficient fibroblasts, consistent with previous data reporting that its expression is specific to testis^30^ and intestine^29^, or induced in response to inflammatory stimuli^31^. To evaluate the effect of *COXFA4* splice site variants on protein levels, COXFA4 protein expression was measured by immunoblotting. Due to high sequence identity (∼61%) and similarity (∼74%) between COXFA4 and its paralog COXFA4L2 (Fig. 3C) and their similar predicted molecular weights (9.4 vs 10 kDa), commercially available antibodies used were unable to fully discriminate between the two proteins, as evidenced by the two closely migrating bands at approximately 10 kDa (Fig. 3D, E). Immunoblotting with an antibody reported to recognise COXFA4 showed a complete loss of the lower molecular weight (MW) band corresponding to COXFA4 in all patient samples, consistent with the absence of *COXFA4* mRNA (Fig. 3D). Interestingly, a higher MW band was detected in all patient-derived samples, particularly in S3, suggesting increased expression of COXFA4L2. To validate this finding, we used a commercially available antibody reported to recognise COXFA4L2 (Proteintech, 16480-1-AP), which also reacts with COXFA4. This antibody detected a higher-MW band in all *COXFA4*-deficient samples and, at lower abundance, also in controls (Fig. 3E), consistent with basal COXFA4L2 expression in fibroblasts and its upregulation upon COXFA4 loss. To further confirm the identity of the higher MW band, a transient siRNA-mediated knockdown of COXFA4L2 was performed in control and *COXFA4*-deficient fibroblasts from S5 (Fig. 3F) and S3 (Supplementary Fig. 5A). Knockdown resulted in ~70% reduction in the intensity of the upper band in the patient-derived fibroblasts, confirming its identity as COXFA4L2. Moreover, these findings show that both COXFA4 and COXFA4L2 are co-expressed in human skin fibroblasts, although COXFA4L2 is present at levels that are barely detectable by immunoblot in control lines. To determine whether COXFA4L2 upregulation was a general response to impaired COX enzyme activity and/or assembly, we analysed a patient-derived cell line carrying a pathogenic variant in *SURF1*, a gene involved in COX assembly and associated with a Leigh-like phenotype^34^. Despite the significant COX deficiency previously reported in *SURF1*-mutant cells^35^, no increase in COXFA4L2 expression was observed (Supplementary Fig. 5B). This suggests that the upregulation of COXFA4L2 is specifically induced by COXFA4 loss, rather than being a general consequence of COX dysfunction.

To further explore the role of COXFA4L2, we assessed the stability of its association with the COX holoenzyme under increasing detergent concentrations. Previous studies have shown that COXFA4 binds weakly to COX and that its detection is highly sensitive to the detergent conditions used during sample preparation^23^. To determine whether COXFA4L2 was associated with the COX complex in a similar manner, n-dodecyl-β-d-maltoside (DDM) titration was performed in control and patient-derived fibroblast (S4 and S5), followed by blue-native PAGE (BN-PAGE) and immunoblotting for COX (Fig. 3G and Supplementary Fig. 5C). In control cells, COXFA4 dissociated from the COX complex at DDM concentrations above 0.02%, consistent with previous findings^23^. In contrast, COXFA4-deficient cells showed an increased COX complex signal at higher DDM concentrations ( > 0.04%), indicating that COXFA4L2 was more stably associated with the complex under the same conditions. This biochemical finding was further supported by in silico structural modelling (Fig. 3H and Supplementary Tables 1 and 2). To compare the affinities of COXFA4 and COXFA4L2 for the COX complex, we generated a comparative 3D model of COXFA4L2 using SwissPDBViewer, based on the structure of COXFA4 (61% sequence identity; chain N, PDB ID: 5Z62). The COXFA4L2 model was then superimposed onto the native COXFA4 position within the COX complex, and both structures were embedded into a lipid bilayer and subjected to energy minimisation. The resulting structures showed a low RMSD (Root Mean Square Deviation) of 0.8 Å, indicating minimal structural perturbation. Protein–protein interface analysis using FoldX revealed that the COX complex containing COXFA4L2 engaged in additional short-range interactions ( < 4 Å) involving residues Q178 (COX1), T98 (COX5B), and W31 (COX6B1), which were absent in the native COXFA4-containing complex (Supplementary Table 1). Additionally, the COXFA4L2-containing model exhibited a slightly more favourable interaction energy, suggesting a modestly enhanced binding affinity and structural stability compared to COXFA4 (Supplementary Table 2). Together, the DDM and in silico data suggest that splice-site variants disrupting COXFA4 expression are partly compensated by upregulation of COXFA4L2. This increase likely helps to partially preserve COX assembly and function in the absence of COXFA4.

### Variants in *COXFA4* affect COX enzymatic activity

**Fig. 4:**
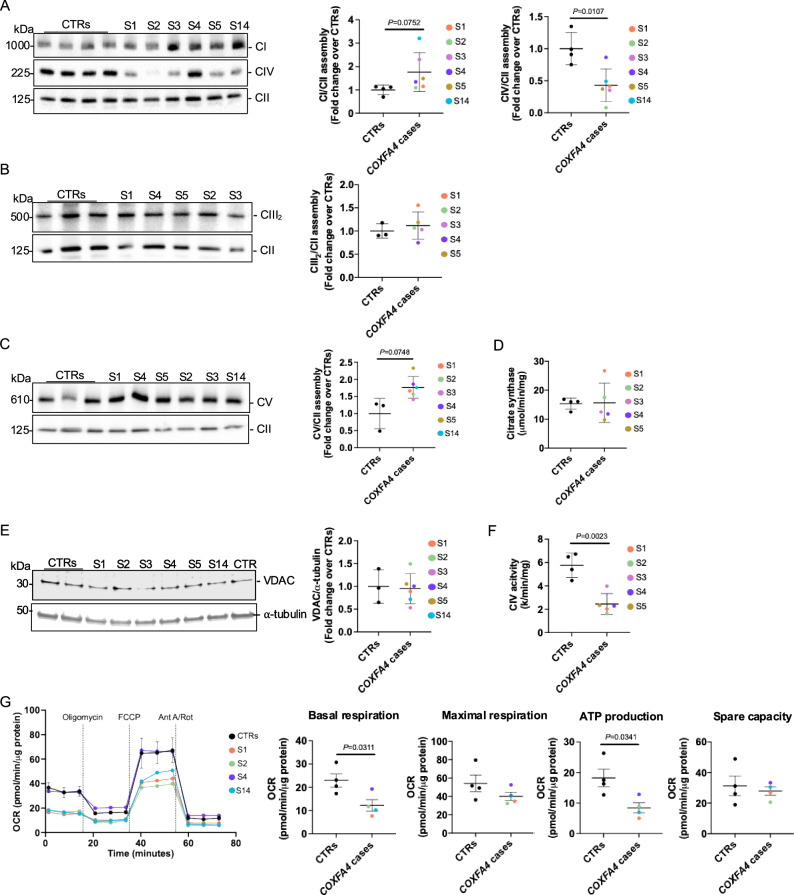
Mitochondrial respiratory chain analysis in *COXFA4* patient-derived primary fibroblasts. **A** Blue-native PAGE (BN-PAGE) analysis of mitochondria from four healthy controls (CTRs) and six *COXFA4* patients (S1–S5 and S14), showing fully assembled complex I (CI), complex IV (CIV) and complex II (CII). Complex II was used as a loading control. **B** BN-PAGE analysis of fully assembled complex III_2_ (CIII_2_) and complex II in mitochondria extracted from three healthy controls (CTRs) and five *COXFA4* patients (S1–S5), with complex II as a loading control. **C** BN-PAGE analysis of fully assembled complex V (CV) and complex II (CII) in mitochondria extracted from three healthy controls (CTRs) and six *COXFA4* patients (S1–S5 and S14), with complex II as a loading control. **D** Citrate synthase activity in mitochondrial protein pellets from four healthy controls (CTRs) and five *COXFA4* patients (S1–S5). **E** SDS-PAGE analysis of VDAC protein levels in total cellular protein extracts from three healthy controls (CTRs) and six *COXFA4* patients (S1–S5, S14). **F** Spectrophotometric measurement of complex IV (CIV) enzymatic activity in mitochondrial protein pellets from four healthy controls (CTRs) and five *COXFA4* patients (S1–S5). **G** Oxygen consumption rate (OCR) in intact primary fibroblasts from four healthy controls (CTRs) and four *COXFA4* patients (S1, S2, S4, and S14), measured using sequential injections of oligomycin, FCCP, and antimycin A/rotenone (AntA/Rot). Error bars indicate SD and data are presented as mean ± SD. Each data point represents an independent biological sample. Statistical significance was assessed using an unpaired two-tailed Welch’s t-test.

To investigate the impact of COXFA4 deficiency on the assembly of ‘free’ OXPHOS complexes, we performed BN-PAGE on DDM-treated mitochondria isolated from *COXFA4* patient-derived cells (S1-S5 and S14). This analysis revealed a reduction in fully assembled complex IV in all *COXFA4*-deficient samples (Fig. 4A). Complex III_2_ assembly was unaffected, whereas complex I levels varied across samples, with several cases showing increased abundance relative to controls (Fig. 4A, B). Complex V assembly was also increased in COXFA4-deficient cells (Fig. 4C), suggesting a compensatory response to maintain ATP production. Mitochondrial mass, assessed by citrate synthase activity, was comparable between patient and control cells (Fig. 4D). Similarly, steady-state levels of other mitochondrial proteins, including VDAC and SDHA, showed no differences between patient and control samples (Fig. 4E and Supplementary Fig. 5D). Consistent with the observed decrease in COX assembly, spectrophotometric measurements of *COXFA4-*deficient samples showed a 60–85% reduction complex IV enzymatic activity (Fig. 4F). Bioenergetic impairment was further confirmed by mitochondrial respiration analysis using the Seahorse flux assay in fibroblasts derived from cases S1, S2, S4 and S14, which showed a significant decrease in basal respiration (∼50%) and ATP-linked oxygen consumption (∼46%) in all samples (Fig. 4G). Importantly, the steady-state levels of subunits from other OXPHOS complexes (Supplementary Fig 5E) and mtDNA relative abundance (Supplementary Fig 5F) were not altered. In contrast, we observed a trend towards reduced levels of COX subunits associated with the COX2 and COX3 modules (Supplementary Fig. 6A, B). Overall, these results indicate that the effects of COXFA4 loss are specific to COX assembly and activity

**Fig. 5:**
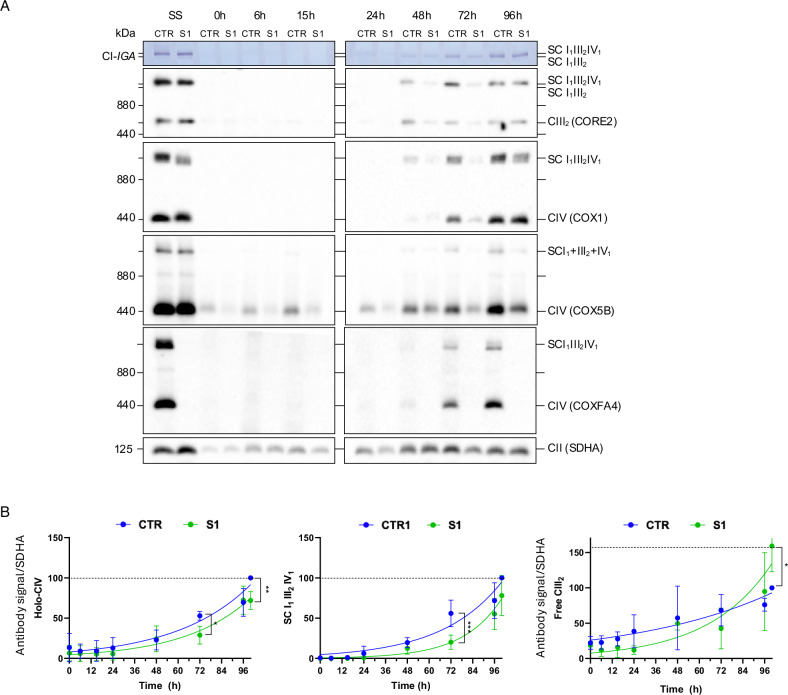
Kinetics of mitochondrial respiratory complex and supercomplex assembly measured in isolated mitochondria from immortalised fibroblasts. **A** Representative blue-native polyacrylamide gel electrophoresis (BN-PAGE) analysis from control (CTR) and *COXFA4* patient-derived immortalised fibroblasts (S1), showing the kinetics of mitochondrial respiratory chain complexes reassembly. The reappearance of complexes I (CI), II (CII), III_2_ (CIII_2_), and IV (CIV) over time following doxycycline removal is shown, along with visible supercomplexes (SC) and free complexes: SCI_1_III_2_IV_1_ (respirasome), SCI_1_III_2_, CIII_2_, CIV_1_ and CII. **B** Quantification of incorporation rates for complex I in-gel activity (CI-*IGA*) into the SCI_1_III_2_IV_1_, CORE2 (CIII₂ subunit) into free CIII₂, and CIV subunits COX1, COX5B, and COXFA4 (the latter only in control) into holo-CIV and SCI_1_III_2_IV_1_. COXFA4 was detected using the Abcam antibody (ab129752), which does not reliably detect COXFA4L2 at low basal levels. Densitometric analysis was performed across three independent experiments. Signal intensities for CI-*IGA*, CORE2, COX1, COX5B, and COXFA4 in their respective complexes at the indicated recovery time points (0, 6, 15, 24, 48, 72, and 96 hours) were first normalised to complex II (SDHA) as a loading control and expressed relative to steady-state (SS) levels, set at 100% (dotted line). Normalised data were fitted to an exponential curve, and the results were expressed as mean ± SEM. Statistical comparison between CTR and S1 was performed using two-way ANOVA followed by Tukey’s multiple comparisons test. Exact adjusted *p*-values for the annotated comparisons in panel B are: holo-CIV at 72 h, *p* = 0.024; holo-CIV at 96 h, *p* = 0.0032; SCI_1_III_2_IV_1_ at 72 h, *p* = 0.0003; free CIII_**2**_ at 96 h, *p* = 0.0363.

To further understand the impact of the loss of COXFA4 on the assembly state of OXPHOS complexes and SCs, we analysed digitonin-treated mitochondria by BN-PAGE (Supplementary Fig 6C) in immortalised patient-derived fibroblasts from S1 and S14. In control fibroblasts, COXFA4 showed the typical profile of other COX subunits, being visible in the ‘free’ COX holoenzyme (IV_1_) and respirasome (SCI_1_III_2_IV_1_), while only barely detectable in the complex IV dimer (IV_2_) and SC III_2_IV_1_. Consistent with our previous results (Fig. 4A), BN-PAGE analysis revealed significantly reduced steady-state levels of fully assembled COX, particularly within the respirasome (SC I_1_III_2_IV_1_) and to a lesser extent in free complex IV_1_, in COXFA4-deficient cells (Supplementary Fig. 6C–E). Notably, an increase in free complex III dimer (III₂) was also detected, likely reflecting reduced incorporation of this complex into respirasome (Supplementary Fig. 6F). To directly assess whether loss of COXFA4 alters the kinetics of COX biogenesis and SCs formation, we performed a doxycycline pulse–chase assay to reversibly inhibit mitochondrial translation. Immortalised control and COXFA4-deficient fibroblasts were treated with doxycycline for six days to deplete mtDNA-encoded OXPHOS subunits, followed by doxycycline washout (time 0 h) to allow recovery of mitochondrial translation and de novo assembly. At defined time points after washout, mitochondria were isolated and analysed by BN-PAGE (Fig. 5A) to monitor recovery of ‘free’ OXPHOS complexes and respiratory SCs, and by SDS–PAGE to follow re-accumulation of representative subunits (Supplementary Fig. 7A). In control fibroblasts, COXFA4 reappeared with kinetics similar to those of COX2 and COX3 modules; however, in the absence of COXFA4, COX2 and COX3 subunits reappeared to lower steady-state levels (Supplementary Fig. 7B). Quantitative analysis showed that holo-complex IV levels were significantly reduced in S1 cells at late timepoints (72–96 h), despite comparable half-recovery times (t₅₀ =77.5 h for CTR and 77.8 h for S1; Fig. 5B). Consistently, respirasome reassembly was delayed in S1 cells (t₅₀ = 84.9 h) compared to controls (t₅₀ = 68.3 h), likely reflecting reduced availability of fully assembled complex IV for SC formation. Importantly, COXFA4L2 was not detected in these assays. This is consistent with the very low basal expression of COXFA4L2 in S1 fibroblasts (Fig. 3E) and with the use of the Abcam anti-COXFA4 antibody (ab129752), which, under our experimental conditions, preferentially detects COXFA4. In conclusion, our data show that COXFA4 acts at the late stages of COX biogenesis, contributing to the stability and activity of the COX holoenzyme, consistent with recent structural evidence that COXFA4 is incorporated during the final maturation of CIV and respirasome assembly^36^. The observation that all COX-containing assemblies still form, although at lower levels, is consistent with this late-stage role. As a consequence, the associated changes in SC abundance are most likely secondary to reduced COX holoenzyme accumulation. Overall, loss of COXFA4 affects the stability and steady-state levels of the COX holoenzyme, leading to reduced respirasome abundance and impaired OXPHOS function.

**Fig. 6:**
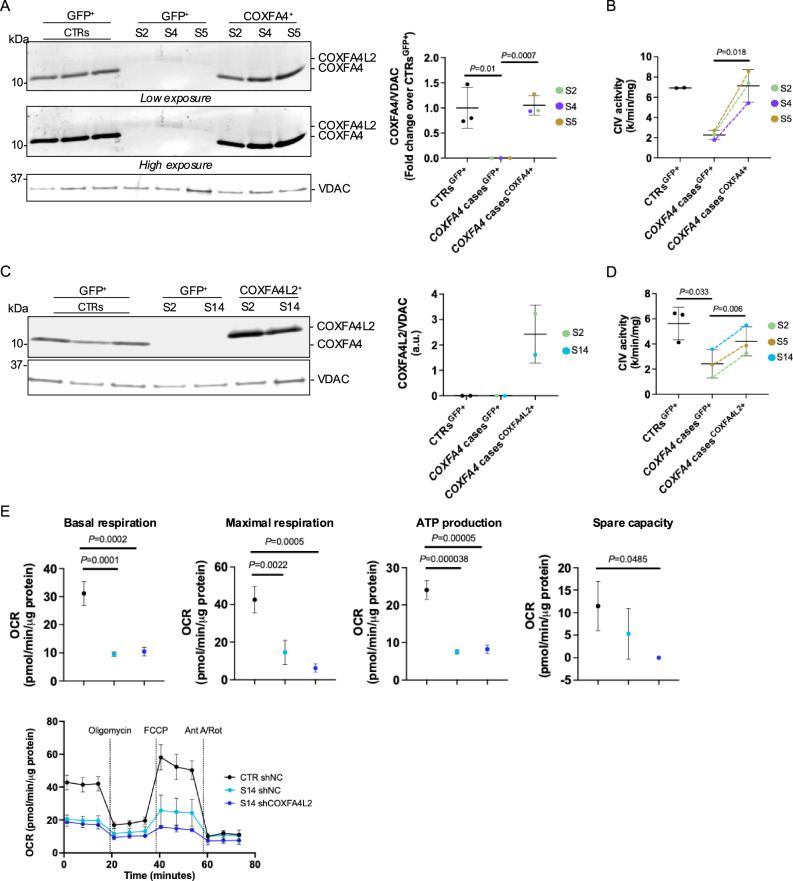
*COXFA4* gene rescue in *COXFA4* patient-derived primary fibroblasts. **A** SDS-PAGE analysis of mitochondria from three healthy controls (CTRs) and three COXFA4 patients (S2, S4, and S5), before and after lentiviral transduction with either an empty vector control (pLenti6.3/V5-DEST-GFP; GFP⁺) or a vector expressing wild-type *COXFA4* (pLenti6.3/V5-DEST-COXFA4; COXFA4⁺). COXFA4 was detected using an antibody recognising both COXFA4 and COXFA4L2 (Stratech Scientific, C16821-ABT). VDAC was used as a loading control. COXFA4 protein levels, normalised to VDAC, were compared between GFP⁺ and COXFA4⁺-transduced samples. Data are shown as mean ± SD; n = 3 per group. **B** Spectrophotometric assay of complex IV enzymatic activity in mitochondrial protein pellets from two CTRs and three COXFA4 patient cell lines (S2, S4, and S5), before and after transduction with GFP⁺ or COXFA4⁺ vectors. Control samples were limited (*n* = 2) due to limited availability of control mitochondrial material. Complex IV activity was compared within patient lines before and after complementation using paired two-tailed t-tests. Each data point represents one independent mitochondrial preparation. **C** SDS-PAGE analysis of mitochondria from three CTRs and two COXFA4 patient cell lines (S2 and S14), before and after transduction with either an empty vector control (pLenti6.3/V5-DEST-GFP; GFP⁺) or a vector expressing wild-type *COXFA4L2* (pLenti6.3/V5-DEST-COXFA4L2; COXFA4L2⁺). Proteins were detected using an antibody reported to recognise COXFA4L2 (Proteintech, 16480-1-AP), which also detects COXFA4. VDAC was used as a loading control. **D** Spectrophotometric measurement of complex IV activity in mitochondrial protein pellets from three CTRs and three COXFA4 patient cell lines (S2, S5, and S14), before and after transduction with GFP⁺ or COXFA4L2⁺ vectors. Error bars indicate standard deviation (SD); each data point represents an independent biological sample. Statistical significance relative to CTRs was assessed using an unpaired two-tailed Welch’s t-test. Comparisons within patient cell lines before and after transduction were performed using paired two-tailed t-tests. **E** Oxygen consumption rate (OCR) in control fibroblasts transduced with shRNA negative control (CTR shNC), and in S14 fibroblasts transduced with shRNA negative control (S14 shNC) or shRNA targeting COXFA4L2 (S14 shCOXFA4L2). OCR was measured using sequential injections of oligomycin, FCCP, and antimycin A/rotenone (AntA/Rot). Basal respiration, maximal respiration, ATP production, and spare respiratory capacity are shown in the corresponding quantification plots. Data are shown as mean ± SD from 3 independent transfections per group. For each independent transfection, technical replicate wells were averaged before statistical analysis. Statistical significance was determined by one-way ANOVA followed by Tukey’s multiple comparisons test.

To confirm that the observed COX defects were due to the loss of COXFA4 expression, we performed complementation assays in patient-derived fibroblasts. Stable expression of wild-type *COXFA4* in fibroblasts of S2 (c.131+1 G > C), S4 (c.131+5 G > A), and S5 (c.42+2 T > C, Fig. 6A) significantly restored complex IV activity, resulting in a ~ 3-fold increase compared to uncomplemented patient fibroblasts (Fig. 6B). We next tested whether the paralog COXFA4L2 could substitute for COXFA4 by transducing fibroblasts from S2, S5, and S14 with a wild-type *COXFA4L2* construct (Fig. 6C). Expression of *COXFA4L2* also improved complex IV activity, though less effectively, with a ~ 1.7-fold increase relative to uncomplemented cells (Fig. 6D). Finally, to assess the functional contribution of COXFA4L2 to cellular bioenergetics, we performed shRNA-mediated knockdown in S14 fibroblasts (Fig. 6E). Basal respiration was preserved, whereas maximal respiration was significantly reduced in S14 COXFA4L2-depleted cells relative to S14. This suggests a selective loss of spare respiratory capacity, reflecting a reduced ability of mitochondria to meet increased energetic demands or cellular stress. A reduction in this capacity indicates that residual complex IV activity is sufficient to meet the ATP demand, but COXFA4L2 is required to support the additional respiratory capacity needed under stress or high-demand conditions. These data indicate that loss of COXFA4 compromises COX activity and mitochondrial bioenergetics and that COXFA4L2 overexpression can only partially compensate for this defect.

### Depletion of COXFA4 in neuronal cells induces COXFA4L2 expression

**Fig. 7:**
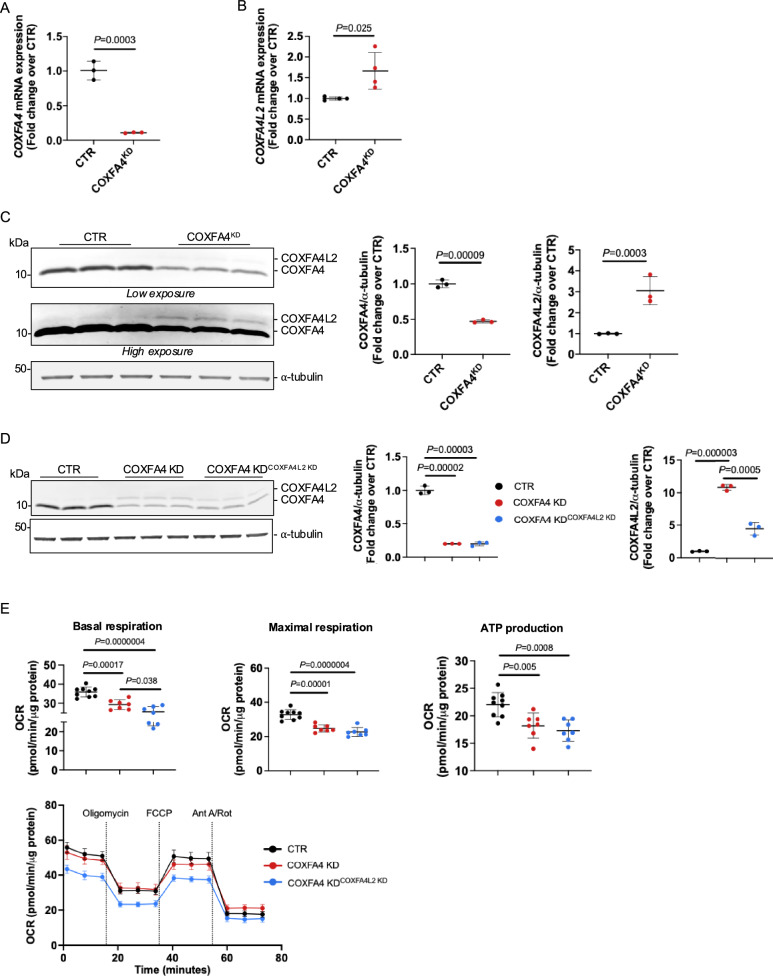
Oxidative phosphorylation analysis in *COXFA4* knockdown SH-SY5Y cells. **A** Quantitative real-time PCR (qPCR) analysis of *COXFA4* mRNA levels following shRNA-mediated *COXFA4* knockdown. **B** qPCR analysis of *COXFA4L2* mRNA levels in the same cells analysed in panel (**A**). In both panels, data are normalised to the housekeeping gene *B2M*. Each data point represents an independent biological replicate (i.e., an independent cell culture); n = 3. Error bars indicate standard deviation (SD). **C** Representative immunoblot showing steady-state protein levels of COXFA4 and COXFA4L2. Membranes were probed with a COXFA4L2 antibody (Proteintech, 16480-1-AP), which also detects COXFA4; α-tubulin was used as a loading control. **D** Immunoblot showing steady-state levels of COXFA4L2 before and after transient knockdown of COXFA4L2 in COXFA4 knockdown cells. Membranes were probed with a COXFA4L2 antibody (Proteintech, 16480-1-AP); α-tubulin was used as a loading control. **E** Oxygen consumption rate (OCR) in SH-SY5Y control cells (CTR), COXFA4-knockdown cells (COXFA4 KD), and double-knockdown cells (COXFA4 KD^COXFA4L2 KD^), measured using sequential injections of oligomycin, FCCP, and antimycin A/rotenone (AntA/Rot). Control (CTR) *n* = 9; COXFA4 KD *n* = 7; and COXFA4 KD^COXFA4L2 KD^ *n* = 7. Data are shown as mean ± SD. Statistical significance was determined by one-way ANOVA followed by Tukey’s multiple comparisons test.

Patients with loss of COXFA4 expression present with a Leigh-like neurodevelopmental disorder associated with brain MRI abnormalities. To confirm our in vitro findings in an independent neuronal-relevant system, we screened five human immortalised cell lines, including neuroglioma (H4) and neuroblastoma (SH-SY5Y) and non-neuronal lines (A549, HeLa, HEK293), for basal COXFA4L2 expression (Supplementary Fig. 8 A). Among these, COXFA4L2 was detectable in SH-SY5Y cells. Although COXFA4L2 is not reported to be constitutively expressed in primary neurons in vivo^37^, the SH-SY5Y cell line is widely used in neurobiology research^38^, and its detectable COXFA4L2 expression provided a unique opportunity to experimentally test the potential compensatory role of this paralog in a neuronal context. We therefore generated a stable COXFA4 knockdown (KD) in SH-SY5Y cells, using a validated shRNA construct (COXFA4^KD3^, Supplementary Fig. 8B). The KD model showed significantly reduced COXFA4 expression at both mRNA (Fig. 7A) and protein levels (Fig. 7C). Notably, COXFA4 KD induced compensatory upregulation of COXFA4L2 mRNA (Fig. 7B) and protein levels (Fig. 7C). This result supports a regulatory mechanism where COXFA4L2 expression is induced in response to COXFA4 deficiency in neuronal cells. To evaluate the functional consequences of COXFA4 depletion in this cellular model, we measured COX activity, which was significantly reduced (∼50%) in COXFA4 KD cells (Supplementary Fig. 8C). The steady-state levels of OXPHOS subunits were not affected (Supplementary Fig. 8D), suggesting that the COX impairment was not due to a general defect in mitochondrial protein expression. To test whether COXFA4L2 upregulation functionally compensates for the loss of COXFA4, we measured mitochondrial oxygen consumption in cells with COXFA4 KD alone and in those with combined KD of COXFA4 and COXFA4L2 (Fig. 7D). Consistent with a compensatory role, the double KD resulted in a more pronounced reduction in basal respiration compared to COXFA4 KD alone (Fig. 7E). Together, these data indicate that loss of COXFA4 induces COXFA4L2 expression as a compensatory mechanism to help maintain residual COX activity and mitochondrial function in neuronal cells.

## Discussion

In this study, we report 12 unrelated families with genetically confirmed *COXFA4*-related PMD, representing the largest documented complex IV-deficient cohort attributable to a single nuclear-encoded structural subunit (Supplementary Table 3), and significantly expanding the known genetic and phenotypic spectrum of COX deficiencies. We identified seven novel biallelic *COXFA4* variants, including five that disrupt mRNA splicing and two that result in partial or complete gene deletions, all leading to complete loss of COXFA4 protein. Clinically, affected individuals exhibited a neurodevelopmental phenotype characterised by early-onset hypotonia, progressive motor dysfunction, spasticity, and varying degrees of intellectual disability, consistent with a Leigh-like encephalopathy, as previously reported^23,33^. These findings broaden our understanding of *COXFA4*-related disease and highlight the essential role of COXFA4 in mitochondrial function and central nervous system development. Emerging evidence implicates COXFA4 in cardiac development^39^. A recent in vivo study in *Xenopus tropicalis* demonstrated that *coxfa4* loss results in craniofacial abnormalities and cardiac defects, including impaired cardiac looping and reduced output^39^. Consistent with a role in cardiac function, we observed ventricular hypertrophy and conduction abnormalities in five individuals (S1–3, S5, and S7), suggesting that cardiac involvement may be subclinical or age-dependent. Importantly, cardiac manifestations are not considered a canonical feature of Leigh syndrome, and they have been reported in ~15–19% of cases^40,41^. Craniofacial anomalies were detected in just three cases (S8, S11, S12), further highlighting the phenotypic diversity associated with COXFA4 deficiency.

Among isolated respiratory chain complex deficiencies, pathogenic variants affecting complex I are the most prevalent ( ~30%). COX deficiencies are relatively rare, accounting for ~8% of reported cases^42,43^. This disparity likely reflects the crucial role of COX as the terminal and rate-limiting complex of the electron transport chain, thus even partial loss of function is poorly tolerated. Consequently, pathogenic variants in nuclear-encoded COX subunits are uncommon and often associated with severe, early-onset phenotypes^10^. However, the identification of the largest known cohort of individuals with biallelic *COXFA4* variants challenges this paradigm. Unlike other nuclear-encoded COX subunits, complete loss of COXFA4 appears to be compatible with life (Supplementary Table 3). Our biochemical data support a role for COXFA4 in late-stage stabilisation of the COX holoenzyme, with secondary consequences for SC abundance. Consistent with this, in situ cryo-EM shows that mammalian COXFA4 is incorporated into all four SCs^25^. These observations support a model in which COXFA4 modulates COX activity and stability, rather than acting as an essential structural component, potentially explaining the relative tolerance of this subunit to pathogenic variants.

Despite the absence of detectable COXFA4 protein, ~30% residual COX activity was retained in mitochondria isolated from patient-derived cultured skin fibroblasts, consistent with previously reported cases^23,33^. Earlier studies suggested that this residual activity might be due to low levels of correctly spliced *COXFA4* mRNA, producing minimal amounts of protein below the detection limit of immunoblotting^23^. However, our transcriptomic data did not support this hypothesis, as only two cases had minor transcript leakage (3.6%-13.8%). Instead, we identified consistent upregulation of COXFA4L2, a poorly characterised paralog of COXFA4 that is expressed at minimal levels under basal conditions in most tissues^29^, suggesting a potential compensatory mechanism. This was supported by functional assays in patient-derived fibroblasts, in which overexpression of COXFA4L2 increased COX activity, albeit less efficiently than COXFA4. This partial rescue suggests that COXFA4L2 can only partly substitute for COXFA4.

The range of clinical severity in our cohort highlights clear differences between individuals. For example, Patient S3 had a milder disease course, higher COXFA4L2 expression, and greater residual COX activity. Although these features occur together, a causal relationship remains to be determined. Individual variability in COXFA4L2 expression in disease-relevant tissues, and the molecular mechanisms underlying this response, will be an important focus of future studies. It also remains to be determined whether other compensatory pathways are activated in parallel. We observed increased complex V assembly in COXFA4-deficient fibroblasts, consistent with a broader adaptive response to OXPHOS dysfunction. Similar changes are reported in other models of complex IV dysfunction, consistent with a metabolic adaptation to OXPHOS impairment^44,45^.

Compensatory responses have been reported in patient-derived models for COX4I1/2^46^ and suggested for COX6A1/2, both of which retain partial COX activity. Similarly, the COX7A1/2 and COX7A2/COX7A2L switch has also been demonstrated in immortalised human cell and mouse models^47–49^. However, no compensatory upregulation of COX8C was reported in *COX8A*-deficient patient-derived fibroblasts, probably due to the tissue-specific expression of these isoforms. For example, COX4I2 is predominantly expressed in the lung, COX6A2 and COX7A1 are muscle-specific, and COX8C is enriched in the heart and brain^22^. Therefore, the absence of these isoforms in patient-derived fibroblasts, as reported for *COX8A* cases, might be a limitation of the in vitro models, which fail to recapitulate the molecular consequences of COX pathogenic variants.

One point not addressed in this work is the mechanism by which COXFA4L2 transcription is induced in the absence of the COXFA4. COXFA4L2 has been reported to be upregulated by HIF‑1α^32^; however, we did not observe elevated HIF-1α at either the transcript or protein level in the COXFA4-deficient fibroblasts. This suggests that either HIF-1α is rapidly degraded in normoxic conditions^50,51^, or *COXFA4L2* induction may occur via an alternative HIF-1α-independent pathway. Importantly, COXFA4L2 is constitutively expressed in brain pericytes, but not in astrocytes or neurons^37^. We detected COXFA4L2 upregulation in SH-SY5Y neuroblastoma cells following COXFA4 knockdown, indicating that this line has the capacity to activate the paralog in the absence of COXFA4. Although SH-SY5Y cells do not fully recapitulate primary neuronal biology, they provide a useful neuronal-like system to test the compensatory potential of COXFA4L2. Whether primary neurons or other neural cell types can trigger a similar response in vivo remains unclear. Future work will be required to define transcriptional regulators responsible for COXFA4L2 expression in normoxia and to clarify its role in different neural and non-neural cell types. In addition to hypoxia-related regulation, paralog switching has also been linked to inflammatory signalling. For instance, COXFA4L3 is induced by proinflammatory stimuli in macrophages, replacing COXFA4^31^. Although we did not detect COXFA4L3 expression in our models, we cannot rule out that stress- or inflammation-related pathways may influence COXFA4L2 regulation in specific tissues. This will need to be explored in future studies using more disease-relevant cell models.

From a therapeutic perspective, the most direct approach would be to restore COXFA4 expression using AAV-based gene replacement. This strategy is supported by our complementation experiments, in which re-expression of wild-type COXFA4 increased COX activity in patient-derived fibroblasts. Strategies based on COXFA4L2 may be considered complementary, since its overexpression only partially restored COX activity, yet still improved residual function, suggesting that it could be useful when gene replacement is not feasible. For example, approaches that enhance endogenous COXFA4L2 expression or promote its stable incorporation into the COX holoenzyme could provide a paralog-based therapeutic avenue for COX-related PMDs.

In this study, we identify COXFA4 as a late-stage assembly subunit critical for COX activity and stability and show that COXFA4L2 upregulation may act as a potential compensatory response to COXFA4 loss. The partial paralog compensation and non-essential role of COXFA4 in COX assembly may explain the relatively high viability of affected individuals. The identification of additional cases and tissue-specific functional studies will be essential to better define genotype–phenotype relationships and to clarify how the expression of COX isoforms is regulated. These insights may inform the development of therapeutic strategies based on paralogs modulation for COX-related PMDs.

## Methods

### Identification and recruitment of affected individuals

Families with biallelic variants in *COXFA4* were identified using several platforms, including GeneMatcher, the RD-Connect Genome-Phenome Analysis Platform (GPAP), the 100,000 Genomes Project^52^, NHS England Genomic Medicine Service and data sharing with collaborators. Written informed consent for genetic testing and data sharing was obtained from all participants or their legal guardians, in accordance with the Declaration of Helsinki via several research studies depending on the individual’s country of origin. Specifically, the Medical Research Council (UK) International Centre for Genomic Medicine in Neuromuscular Diseases (ICGNMD) was approved by the relevant Research Ethics Committee (REC) [London—Camberwell St Giles REC (REC ref. 19/LO/1796)]. The 100,000 Genomes Project was approved by the relevant REC [East of England—Cambridge South (REC ref. 14/EE/1112)], and all participants provided informed consent. The study was also approved by the REC at the Institute of Neurology University College London (REC ref. 09/H0716/76). The Department of Clinical Research and Development Public Assistance Hospitals of Paris, in connection with the Ministry of Higher Education and Research located in Paris, France, approved skin fibroblast harvest, research use, and conservation in a research biorepository maintained in Bicêtre Hospital, Paris, France, under declaration DC 2009-939. The clinical and genetic details of all individuals are provided in Tables 1 and 2.

### Whole-genome and whole-exome sequencing

Variants in the *COXFA4* gene were identified in all affected individuals using whole-exome sequencing (WES) or whole-genome sequencing (WGS). Sequencing data were processed, and variants were filtered and prioritised using in-house bioinformatics pipelines at the respective contributing centres. Candidate variants were analysed using the Integrative Genomics Viewer (IGV) and confirmed by Sanger sequencing in all families.

### Cell culture

Primary and immortalised patient-derived fibroblasts and H4 cells (American Type Culture Collection, ATCC) were cultured in high-glucose Dulbecco’s Modified Eagle Medium (DMEM, Thermo Fisher Scientific). SH-SY5Y cells (ATCC) were maintained in DMEM-Nutrient Mixture F-12 (DMEM-F12, Thermo Fisher Scientific). All media were supplemented with 4 mM L-glutamine, 110 mg/L pyruvate, 10% (v/v) fetal bovine serum (Gibco, Thermo Fisher Scientific), 100 U/ml penicillin/streptomycin (Gibco, Thermo Fisher Scientific). Cells were incubated at 37 °C under standard conditions (5% CO_2_; atmospheric O₂; 95% relative humidity) and were regularly tested for mycoplasma contamination.

To follow the assembly kinetics of mitochondrial respiratory complexes and supercomplexes, cells were treated with doxycycline, a reversible inhibitor of mitochondrial translation, as previously reported^53^. Briefly, immortalised patient-derived fibroblasts were cultured for 6 days in the presence of 15 µg/ml doxycycline, then washed and cultured in doxycycline-free medium (washout = time 0). Cells were subsequently collected at 0, 6, 15, 24, 48, 72, and 96 h after doxycycline removal. Untreated cells served as positive controls.

### Immortalisation of patient-derived fibroblasts

To immortalise patient-derived fibroblasts, HEK-293T cells were transiently transfected with pLOX-Ttag-iresTK (Addgene plasmid # 12246), together with packaging psPAX2 (#12260, Addgene) and envelope pMD2.G (#12259, Addgene) vectors using Lipofectamine 3000 (Thermo Fisher Scientific) in OptiMEM (Invitrogen). After 18 h, media was replaced with fresh media. Viral particles were collected and filtered 48 h post-transfection, and both patient and healthy control fibroblasts were transduced. Immortalisation was confirmed by long-term culture ( > 10 passages) and monitoring cell proliferation.

### Generation of complemented primary patient-derived fibroblasts

To complement the primary patient-derived fibroblasts, HEK-293T cells were transiently transfected with the target vector, together with packaging psPAX2 (#12260, Addgene) and envelope pMD2.G (#12259, Addgene) vectors using Lipofectamine 3000 (Thermo Fisher Scientific) in OptiMEM (Invitrogen). After 18 h, media was replaced with fresh media. Viral particles were collected and filtered 48 h post-transfection, and both patient and healthy control fibroblasts were transduced with viral particles carrying either the pLenti6.3/V5-DEST-GFP (#40125, Addgene) or the pLenti6.3/V5-DEST vector expressing human *COXFA4* or *COXFA4L2*. After 24 h, the fibroblasts were washed and selected using 4 µg/ml blasticidin (Thermo Fisher Scientific).

### Generation of knockdown (KD) cell lines

SH-SY5Y and H4 cells were stably transduced with lentiviral vectors expressing GIPZ shRNAs targeting *COXFA4*, while patient-derived skin fibroblasts were transduced with GIPZ shRNAs targeting *COXFA4L2* (Horizon Discovery). For each gene, three independent shRNA constructs were evaluated to identify the vector that achieved the most efficient protein knockdown. Specifically, the following shRNAs were screened: COXFA4 (V2LHS_152169, V3LHS_380399, V3LHS_411475) and COXFA4L2 (V3LHS_333562, V3LHS_333564, V3LHS_333563). Lentiviral particles were produced in HEK293T cells by co-transfecting the shRNA expression vector with the packaging plasmid psPAX2 (Addgene #12260) and envelope plasmid pMD2.G (Addgene #12259). Viral supernatants were harvested 48 h post-transfection and used to transduce SH-SY5Y or H4 cells seeded at 70-80% confluence. 24 h after transduction, cells were selected for puromycin resistance. Cells were maintained in 1 μg/ml puromycin until the day before the experiment.

For siRNA transient transfection, SH-SY5Y or patient-derived fibroblasts were seeded at 70–80% confluence and transfected with 5 nM Silencer Select *COXFA4L2* siRNA (Assay ID # s32350, Thermo Fisher Scientific) or Silencer Select Negative control siRNA (Cat # 4390843, Thermo Fisher Scientific) using Lipofectamine 3000 in OptiMEM (Invitrogen) for 6 days (two consecutive transfections every three days).

### Cloning

The plasmid used to complement patient-derived fibroblasts with human *COXFA4* and *COXFA4L2* were generated by Gateway cloning. Briefly, ~200 ng of human *COXFA4* cDNA (MHS6278-202841615, Horizon Discovery) or *COXFA4L2* cDNA (MHS6278-202831227, Horizon Discovery) was used as a template and amplified using Phusion Plus high-fidelity DNA polymerase (Thermo Fisher Scientific) according to the manufacturer’s protocol using the following primers: COXFA4 Fw 5’-AAAAAGCAGGCTATGCTCCGCCAGATCATCGGT-3’ and COXFA4 Rv 5’-AGAAAGCTGGGTTTAGAAATCTGGACGTTCCTT-3’ and COXFA4L2 Fw 5’-AAAAAGCAGGCTATGGCAGGAGCCAGTCTTGGGG-3’ and COXFA4L2 Rv 5’-AGAAAGCTGGGTTTAGAAGTCTGGCCGGTCC-3’. The resulting PCR products were subsequently amplified to add *attB* overhangs using primers 5’-GGGACAAGTTTGTACAAAAAAGCAGGCT-3’ and 5’-GGGGACCACTTTGTACAAGAAAGCTGGGT-3’. The *attB*-flanked PCR products were purified via gel extraction (Monarch DNA Gel Extraction Kit, New England Biolabs and cloned into the pDONOR221 vector using the Gateway BP Clonase II Enzyme mix (Thermo Fisher Scientific). After transformation into DH5α competent *E. coli* cells (Thermo Fisher Scientific), the sequence was verified by Sanger sequencing. The entry clone was transferred into the pLenti6/V5-DEST Gateway Vector (Thermo Fisher Scientific) using the Gateway LR Clonase II Enzyme mix (Thermo Fisher Scientific), followed by transformation and sequencing to confirm the sequence. All primers were purchased from Integrated DNA Technologies (IDT).

### RNA extraction, cDNA synthesis, and RT-qPCR

RNA was extracted from cell pellets using the RNeasy Mini Kit (Qiagen), followed by the removal of genomic DNA contamination using the DNA-free DNA Removal Kit (Thermo Fisher Scientific). cDNA was synthesised using the High-Capacity cDNA Reverse Transcription Kit (Thermo Fisher Scientific). Gene expression was measured using TaqMan Fast Advanced Master Mix (Thermo Fisher Scientific), following the manufacturer’s instructions. Expression levels were determined using the ΔΔCt method^54^, and all experiments were performed in technical triplicates. Gene expression data were normalised to the *B2M* reference gene.

The following primer/probes used were purchased from IDT:

B2M probe (5’-/5HEX/ATGTGTCTGGGTTTCATCCATCCGACA-3’)

B2M primer 1 (5’-CCAGCAGAGAATGGAAAGTCAA-3’)

B2M primer 2 (5’-TCTCTCTCCATTCTTCAGTAAGTCAACT-3’)

COXFA4 probe (5’-/5FAM/ACCCAGTTTGTTCCAGGGCTCT-3’)

COXFA4 primer 1 (5’-GCATTGTTCAATCCAGATGTTTG-3’)

COXFA4 primer 2 (5’-GCTTGCTGTAATCCACATTCAC -3’)

COXFA4L2 probe (5’-/5FAM/TTGTTCTTTCTGTCCCAGCAGACGTC -3’)

COXFA4L2 primer 1 (5’-GCTGCGCTTTACTTGCTG-3’)

COXFA4L2 primer 2 (5’-AAGGAACTTGTATTGGTCATTGG -3’)

### RNA sequencing

RNA was isolated from patient-derived fibroblasts and sequenced on an Illumina NovaSeq with 100 bp paired-end reads following library preparation using Kapa mRNA Hyper Prep Sequencing (UCL Genomics, London). Samples were sequenced to a depth of 100 million reads. FASTQ files were aligned using Spliced Transcripts Alignment to a Reference (STAR) software^55^. QC was performed via MultiQC on STAR and FastQC data^56^. BAM files and Sashimi plots were visualised using Integrative Genomics Viewer (IGV). Differential analysis on pseudoaligned data was performed using the Sleuth package, followed by processing using Kallisto^57^.

### RT-PCR, Sanger sequencing and genomic array analysis

Confirmation of *COXFA4* c.42+1del, c.42+2 T > C, c.42+1 G > C, c.131+1 G > C, and c.131+5 G > A variants was assessed using standard PCR-based sequencing. DNA was amplified using Phusion Plus DNA Polymerase (Thermo Fisher Scientific), according to the manufacturer’s protocol and PCR products were run on a 1.2% agarose gel, isolated using the Monarch DNA Gel Extraction Kit (New England BioLabs) and sent for Sanger sequencing analysis (Azenta Life Sciences). All primers were purchased from IDT:

COXFA4 c.42 Fw (5’-GAGGTCCTGGGTGACTTTGG-3’)

COXFA4 c.42 Rv (5’-TGCGGCGAATACAAGAACCT -3’)

COXFA4 c.131 Fw (5’-TGGTTTAGTTGATCCCCCTCT-3’)

COXFA4 c.131 Rv (5’-ACCAAAGAGAAAACGAGACTCAGA -3’)

Genomic array analysis was performed using Infinium Global Diversity Array-8 Kit (Illumina) at UCL Genomics (UCL Great Ormond Street Institute of Child Health, London, UK) following the manufacturer’s instructions. Raw IDAT files were processed using Genome Studio (Illumina) and converted to the PLINK format (https://www.cog-genomics.org/plink/1.9). Homozygosity regions were identified using PLINK and visualised using the Gviz package in R.

### Multiple sequence alignment

COXFA4 (NCBI Reference Sequence: NP_002480.1) and COXFA4L2 (NCBI Reference Sequence: NP_064527.1) protein sequences from *Homo sapiens* were aligned using Clustal Omega. The identical and highly conserved residues are highlighted in green and purple, respectively.

### SDS-PAGE and immunoblotting

Cells were lysed in RIPA buffer (50 mM Tris-HCl pH 7.4, 150 mM NaCl, 0.25% sodium deoxycholate, 1 mM EDTA, and 1% NP-40) supplemented with a 1× complete protease inhibitor cocktail (Roche Molecular Diagnostics). Lysates were resolved on 10–12% Tricine or 16% Tricine polyacrylamide gels (Thermo Fisher Scientific) and transferred to Trans-Blot nitrocellulose membranes (Bio-Rad). Membranes were blocked with Intercept (PBS) Blocking Buffer (Li-Cor Biosciences) and incubated overnight at 4 °C with primary antibodies. Following incubation, membranes were washed three times for 15 min each with TBS containing 0.1% Tween-20 and probed with infrared dye-conjugated secondary antibodies. Primary antibodies included those against COXFA4 (C16821-ABT, Stratech Scientific; 1:1000), COXFA4L2 (16480-1-AP Proteintech; 1:500), VDAC (D73D12, Cell Signalling Technology; 1:1000), OXPHOS human antibody cocktail (ab110411, Abcam; 1:1000), COX4 (MA5-15078, Thermo Fisher Scientific; 1:1000), α-tubulin (66031-1-Ig, Proteintech; 1:10,000), GAPDH (AM4300, Thermo Fisher Scientific; 1:10,000), and β-actin (4970, Cell Signalling Technology; 1:10,000). Secondary antibodies used were IRDye 680LT Goat anti-Mouse IgG (926–68020, Li-Cor Biosciences; 1:10,000) and IRDye 800CW Goat anti-Rabbit IgG (926–32211, Li-Cor Biosciences; 1:10,000). Fluorescent signals were detected using a Li-Cor Odyssey CLx infrared imaging system at 680 nm and 800 nm. Band intensities were quantified using ImageJ v2.0.0 (NIH, USA) and normalised to GAPDH, α-tubulin or β-actin as loading controls.

For the assembly kinetics of mitochondrial respiratory complexes experiments, mitochondrial pellets were isolated from 175 cm² cell culture flasks or 15-cm dishes (Thermo Fisher Scientific) as described in ref. ^58^. Protein concentration was determined using the BCA protein assay kit (Thermo Fisher Scientific). Protein extracts (25 µg) were separated on 10% SDS-PAGE gels and transferred onto PROTAN (Schleicher & Schuell) nitrocellulose membranes using conventional procedures. For both SDS–PAGE and BN-PAGE analyses, band intensities were quantified using Image Lab software (Bio-Rad), background-subtracted, normalised to complex II (SDHA) as a loading and solubilisation control, and expressed relative to steady-state levels, which were set to 100%.

### Crude mitochondrial fractions

Mitochondria were isolated by differential centrifugation from seven confluent T75 flasks of patient-derived fibroblasts or four confluent T75 flasks of SH-SY5Y cells. Briefly, cell pellets were manually homogenised in 500 μl of mitochondrial extraction buffer (10 mM HEPES pH 7.4, 250 mM sucrose, 1 mM sodium EDTA, pH 8.0) supplemented with the protease inhibitors 2 mM phenylmethanesulfonyl fluoride (PMSF), 2 μg/ml pepstatin A and 2 μg/ml leupeptin using a glass Dounce homogeniser. The homogenates were centrifuged at 1500 × *g* for 10 min at 4 °C to remove cell debris and nuclei. The resulting supernatants were collected and stored on ice. The pellets were resuspended in fresh 500 μl of extraction buffer, re-homogenised, and centrifuged at 1500 × *g* for 10 min at 4 °C. The two supernatants were combined and centrifuged at 1500 × *g* for 10 min at 4 °C to remove residual cell debris and nuclei. The clarified supernatants were then transferred to a clean tube and centrifuged at 11,500 × *g* for 12 min at 4 °C to isolate the mitochondrial-enriched pellets. These pellets were resuspended in 80–100 μl of mitochondrial extraction buffer and used for spectrophotometric enzyme assays, in-gel activity staining, and blue-native PAGE. Protein concentrations were determined using Bradford Protein Assay (Thermo Fisher Scientific).

### 3D molecular modelling analysis

A 3D comparative model of COXFA4L2 was generated based on the human COXFA4 structure from the crystal structure of *Homo sapiens* cytochrome *c* oxidase (PDB_ID: 5z62). This cytochrome *c* oxidase is crystallised in complex with one cardiolipin, two heme-A, three molecules of 1,2-dioleoyl-sn-glycero-3-phosphoethanolamine, one zinc ion, three copper ions, and one magnesium ion, as described in ref. ^4^. To propose a structural model of cytochrome *c* oxidase incorporating COXFA4L2 in place of COXFA4, we used the 5z62.pdb structure as a template. The predicted 3D structure of COXFA4L2 was superimposed onto the position of NDUFA4 (namely, COXFA4, chain N of 5z62.pdb) in the crystallized structure, thereby generating a modified model in which COXFA4L2 occupies this position (designated as chain X). Coordinates for the surrounding cofactors and ligands, including cardiolipin, two heme-A, three molecules of 1,2-dioleoyl-sn-glycero-3-phosphoethanolamine one zinc ion, three copper ions, one magnesium, were retained from the 5Z62 structure and transferred during the superimposition process. All structural alignments were performed using the super command implemented in PyMOL 2.5.4, according to established protocols^59,60^. The resulting models of cytochrome *c* oxidase containing either COXFA4 or COXFA4L2 were used for comparative structural analyses.

### Membrane building and energy minimisation

Both the COXFA4L2-containing model and the native crystal structure (PDB ID: 5Z62) were embedded in a model of the mitochondrial inner membrane bilayer composed of 1-palmitoyl-2-oleoyl-sn-glycero-3-phosphocholine (POPC), 1-palmitoyl-2-oleoyl-sn-glycero-3-phosphoethanolamine (POPE), and tetraoleoyl cardiolipin (TOCL2), which are considered the main components of the mitochondrial inner membrane^61,62^. Membrane systems were constructed using Membrane Builder plug-in and CHARMM topology available under the Charmm-GUI Effective Simulation Input Generator^63–65^.

The membrane bilayer was built up as a 150–150 Å side square POPC/POPE/TOCL2 patch for the generated protein complex 3D models. In the setup phase, the psfgen tool was  used to generate a complete all-atom psf file of the system. The obtained structure was solvated in a double layer of TIP3P (transferable intermolecular potential 3P) by setting the water thickness (minimum water height on the top and bottom of the system) to 25 Å. Then, periodic boundary conditions with box dimensions 150 (x) × 150 (y) × 165 (z) Å and PME (particle mesh Ewald) were applied for each investigated cytochrome c oxidase 3D model to compute full electrostatics for the generated systems. To mimic physiological conditions, K⁺ and Cl⁻ ions were added to a final concentration of 200 mM using The Include Ions plug-in with a 5 Å exclusion radius around the protein and a minimum interionic distance of 5 Å, according to previously validated protocols^59,60^. The simulation temperature was kept constant at 300 K by means of a Langevin thermostat, and the pressure was set to 1 atm (101.325 kPa) using a Langevin piston during the minimisation and equilibration protocol, for which an integrator time step was set to 1 fs and the ‘rigidbonds’ parameter was set to ‘all’. Starting from the above-described systems, 50,000 equilibration (conjugate gradient) steps were performed. All the equilibration steps were performed by using NAMD2 with the force field CHARMM36m with cmap correction^63–65^, as previously described^59,60^.

### Protein–protein interaction energy analysis

To evaluate the binding affinity of COXFA4 or COXFA4L2 with the other subunits of the cytochrome *c* oxidase at the protein–protein interface, we calculated protein–protein interaction energies using the FoldX AnalyseComplex assay^60,66,67^. This method estimates binding energy by computationally unfolding the selected targets and determining the stability of the remaining molecules, and then subtracting the sum of the individual energies from the global energy^60,66,67^. More negative energy values indicate stronger, more stable binding interactions, whereas positive energy values indicate no binding^60,66,67^.

### mtDNA quantification

Total DNA was extracted from patient-derived fibroblasts as previously described^68^. The relative abundance of mitochondrial DNA was quantified by multiplex quantitative polymerase chain reaction (qPCR) using TaqMan primer/probe sets targeting mitochondrial *ND1* (VIC-labelled; primer:probe ratio of 1:1) and nuclear *B2M* (FAM-labelled; primer:probe ratio of 3:1). Reactions were performed using TaqMan Fast Advanced Master Mix (Thermo Fisher Scientific) with 4.6 ng of DNA per reaction and 5 μM primer/probes, in a total volume of 10 μL. The relative mtDNA content was calculated using the ΔΔCt method^54^.

### Blue-native polyacrylamide gel electrophoresis (BN-PAGE) and in-gel activity assays

To assess the assembly of oxidative phosphorylation (OXPHOS) complexes by BN-PAGE, mitochondria isolated from patient-derived fibroblasts were resuspended in 100 µL of mitochondrial extraction buffer and lysed through three consecutive freeze-thaw cycles. Protein concentration was determined using the Bradford Protein Assay (Thermo Fisher Scientific) and diluted into an equal protein concentration and 1/6 volume of 0.5% n-dodecyl-β-D-maltoside (DDM), 1 M 6-aminocaproic acid, and 5% Serva blue G (Serva Electrophoresis, 3505003, Heidelberg, Germany). Native protein complexes were resolved using 3%–12% native polyacrylamide gels with a 3% stacking gel (2–3 μg of protein/lane) as previously described^69^.

To determine the optimal concentration of DDM for analysing COXFA4 and COXFA4L2 as components of COX holoenzyme by BN-PAGE, patient-derived total cell pellets were lysed in extraction buffer containing serial dilutions of DDM (0.01%, 0.02%, 0.04%, 0.08%, 0.16%, and 0.32%), 1 M 6-aminocaproic acid, 50 mM bis-tris (pH 7.0), 1 mM PMSF, 1 μg/mL leupeptin, and 1 μg/mL pepstatin A. Samples were incubated on ice for 15 min and centrifuged at 13,000 g for 15 min at 4 °C, protein lysates were added 1/6 volume of 1 M 6-aminocaproic acid, 5% Serva blue G (Serva Electrophoresis, 3505003, Heidelberg, Germany) and native samples were separated using 8–16% native polyacrylamide gels with a 3% stacking gel.

BN-PAGE gels were transferred onto Immobilon-PSQ PVDF membranes (Millipore, 1SEQ00010, Burlington, MA, USA)^70^. Membranes were rinsed three times with methanol to remove residual Serva blue G dye and then blocked with 10% (w/v) skimmed milk powder in PBS for 1 h at room temperature. Membranes were incubated overnight at 4 °C with the following primary antibodies diluted in 3% BSA in PBS containing 0.1% Tween-20 (PBS-T): anti-NDUFS3 (Abcam, ab14711; 1:300), anti-MTCO1 (Abcam, ab14705; 1:3000), anti-UQCRC2 (Abcam, ab14745; 1:1000), anti-SDHA (Abcam, ab14715; 1:6000), anti-ATP5A (Abcam, ab14748; 1:9000), anti-COXFA4L2 (16480-1-AP Proteintech), and anti-COXFA4 (Stratech Scientific, C16821-ABT; 1:1000). Both the Stratech anti-COXFA4 (C16821-ABT) and the Proteintech anti-COXFA4L2 (16480-1-AP) show cross-reactivity toward COXFA4 and COXFA4L2. Band identity was confirmed by siRNA-mediated knockdown of COXFA4L2 (Fig. 3F; and Supplementary Fig. 5A). After primary antibody incubation, membranes were washed in PBS-T and incubated for 1 h at room temperature with anti-rabbit (P0448, Dako, 1:4000) or anti-mouse (W402B, Promega, 1:3000) horseradish peroxidase (HRP)-conjugated secondary antibodies for 1 h. Images were detected with a Bio-Rad ChemiDoc MP Imaging System and quantified using the Bio-Rad Image Lab 5.1 software.

### Assembly kinetics of mitochondrial respiratory complexes

To investigate the assembly kinetics of mitochondrial respiratory complexes and SCs, cells were treated with doxycycline, a reversible inhibitor of mitochondrial translation, as previously reported^53^. Briefly, immortalised patient-derived fibroblasts were cultured for 6 days in the presence of 15 µg/mL doxycycline to deplete mtDNA-encoded OXPHOS subunits, then washed and cultured in doxycycline-free medium (washout = time 0). Cells were subsequently collected at 0, 6, 15, 24, 48, 72, and 96 h after doxycycline removal. Untreated cells cultured in parallel served as positive controls (steady-state, SS).

Mitochondrial pellets were isolated from immortalised patient-derived fibroblasts cultured in P150 cell culture flasks or 15-cm dishes (Thermo Fisher Scientific), as described previously^58^. Unless otherwise indicated, samples were solubilised using digitonin at a detergent-to-protein ratio of 4:1. Pre-cast NativePAGE 3-12% Bis-Tris gels (Invitrogen) were loaded with 40 µg of mitochondrial protein and processed for BN-PAGE, as previously described^53^. After electrophoresis, proteins were transferred to PVDF membranes at 40 V overnight. In parallel, 25 µg of the same mitochondrial extracts were processed for SDS-PAGE, and proteins were transferred to nitrocellulose membranes at 150 V for 2 h s. Membranes were probed with the following primary antibodies: anti-UQCRC2 (Abcam, ab14745), anti-MTCO1 (Abcam, ab14705), anti-MTCO2 (Abcam, ab110258), anti-COX6B (Abcam, ab110266), anti-COX5B (Santa Cruz, sc-374417), anti-COXFA4 (Abcam, ab129752), anti-SDHA (Abcam, ab14715), anti-NDUFA9 (Abcam, ab14713), anti-NDUFV1 (Proteintech, 11238-1-AP), anti-COX4 (Abcam, ab62164), anti-COX6C (Abcam, ab110267). Immunoreactive bands were detected using HRP-conjugated secondary antibodies and ECL Prime Western Blotting Detection Reagent in a ChemiDoc MP Imager (Bio-Rad). For both BN-PAGE and SDS–PAGE analyses, band intensities were normalised to complex II (SDHA) as a loading control, and expressed relative to steady-state levels, which were set to 100%. Normalised data were fitted to an exponential curve, and the results were expressed as mean ± SEM.

### Cytochrome *c* oxidase activity and citrate synthase activity assay

Cytochrome *c* oxidase activity of mitochondria extracted from patient-derived fibroblasts and SH-SY5Y cells was measured in technical quadruplicate, as previously described^71^. The citrate synthase activity of mitochondria isolated from patient-derived fibroblasts and SH-SY5Y cells was assessed following the protocol described in ref. ^71^. Each biological sample was analysed in quadruplicate.

### Mitochondrial respiration

Oxygen consumption was measured using a Seahorse XF96 Extracellular Flux Analyzer (Seahorse Bioscience). Patient-derived fibroblasts and SH-SY5Y cells were seeded at densities of 20,000 and 40,000 cells per well, respectively, into XF96 Cell Culture Microplates one day before the assay. On the day of the experiment, the culture medium was replaced with the XF Assay Medium (Seahorse XF DMEM Medium, 10 mM glucose, 1 mM pyruvate, and 2 mM L-glutamine). The plates were then incubated for 1 h at 37 °C in a non-CO₂ incubator to equilibrate. Following the measurement of basal respiration, the following mitochondrial inhibitors were sequentially injected: oligomycin (5 µM), FCCP (2 µM for patient-derived fibroblasts; 1 µM for SH-SY5Y), and antimycin A/rotenone (0.5 µM/0.5 µM). Oxygen consumption data were normalised to total protein content. Following the assay, the cells were lysed through three freeze-thaw cycles, and total protein concentration was determined using the Bradford Protein Assay (Thermo Fisher Scientific).

### Statistical analyses

Statistical analyses were performed using GraphPad Prism 8 (GraphPad Software, CA, USA). Depending on the experiment, statistical significance was assessed using two-way ANOVA, two-tailed Mann–Whitney test, paired or unpaired two-tailed t-tests, as specified in the corresponding figure legends. Data are presented as mean ± SD unless otherwise indicated; for the kinetic curve-fitting analysis in Fig. 5, data are presented as mean ± SEM. For qPCR experiments, each biological replicate represents the mean of three technical replicates.

### Reporting summary

Further information on research design is available in the Nature Portfolio Reporting Summary linked to this article.

## Supplementary information

Supplementary information

Reporting Summary

Peer Review file

## Source data

Source data

## Data Availability

The authors confirm that the data supporting the findings of this study are available within the article and/or its Supplementary Information. Source data for all immunoblots are provided with the paper. Raw DNA and RNA sequencing data, as well as neuroimaging data from the individuals herein reported, are securely stored in a controlled-access repository at the UCL Institute of Neurology, University College London, London, UK, and are not publicly available as they comprise part of individuals medical records and contain patient identifiable data. Access to these data is subject to ethical restrictions and will be granted only to clinicians and/or researchers who enter into an appropriate research agreement. These data are also available from the corresponding author upon request subject to completion of any required material transfer agreements (MTAs) and compliance with institutional and legal regulations. Data from the National Genomic Research Library (NGRL) used in this research are available within the secure Genomics England Research Environment. Access to NGRL data is restricted to adhere to consent requirements and protect participant privacy. Data used in this research include: 100,000 Genomes rare disease main release dataset (tiering data) accessed via /gel_data_resources/main_programme/tiering_data_rd/GRCh38/ and filtered by gene name. Access to NGRL data is provided to approved researchers who are members of the Genomics England Research Network, subject to institutional access agreements and research project approval under participant-led governance. For more information on data access, visit: https://www.genomicsengland.co.uk/research Source data are provided with this paper.
