# Supplementary material for: COXFA4L2 upregulation preserves residual cytochrome c oxidase activity in COXFA4-related Leigh-like encephalopathy
Source: Nat Commun. Author manuscript; Available in PMC 2026 Sep 11. (PMC13392013; doi:10.1038/s41467-026-73455-9)
Supplement: Supplementary file 2 [file EMS215615-supplement-Reporting_Summary.pdf]

nature portfolio

Corresponding author(s): Micol Falabella
Robert Pitceathly

Last updated by author(s): Jan 14, 2026

# Reporting Summary

Nature Portfolio wishes to improve the reproducibility of the work that we publish. This form provides structure for consistency and transparency in reporting. For further information on Nature Portfolio policies, see our Editorial Policies and the Editorial Policy Checklist.

## Statistics

For all statistical analyses, confirm that the following items are present in the figure legend, table legend, main text, or Methods section.

| n/a | Confirmed | |
|---|---|---|
| ☐ | ☒ | The exact sample size (*n*) for each experimental group/condition, given as a discrete number and unit of measurement |
| ☐ | ☒ | A statement on whether measurements were taken from distinct samples or whether the same sample was measured repeatedly |
| ☐ | ☒ | The statistical test(s) used AND whether they are one- or two-sided<br>*Only common tests should be described solely by name; describe more complex techniques in the Methods section.* |
| ☒ | ☐ | A description of all covariates tested |
| ☐ | ☒ | A description of any assumptions or corrections, such as tests of normality and adjustment for multiple comparisons |
| ☐ | ☒ | A full description of the statistical parameters including central tendency (e.g. means) or other basic estimates (e.g. regression coefficient) AND variation (e.g. standard deviation) or associated estimates of uncertainty (e.g. confidence intervals) |
| ☐ | ☒ | For null hypothesis testing, the test statistic (e.g. *F*, *t*, *r*) with confidence intervals, effect sizes, degrees of freedom and *P* value noted<br>*Give P values as exact values whenever suitable.* |
| ☒ | ☐ | For Bayesian analysis, information on the choice of priors and Markov chain Monte Carlo settings |
| ☒ | ☐ | For hierarchical and complex designs, identification of the appropriate level for tests and full reporting of outcomes |
| ☒ | ☐ | Estimates of effect sizes (e.g. Cohen's *d*, Pearson's *r*), indicating how they were calculated |

*Our web collection on statistics for biologists contains articles on many of the points above.*

## Software and code

Policy information about availability of computer code

| | |
|---|---|
| Data collection | Data were collected using standard laboratory equipment and software |
| Data analysis | Sequence reads (FASTQ files) were aligned using STAR (Spliced Transcripts Alignment to a Reference). Quality control was performed using FastQC and MultiQC. BAM files and Sashimi plots were visualised using Integrative Genomics Viewer (IGV). Pseudoalignment was performed using Kallisto, and differential expression analysis was conducted using the Sleuth package. Additional statistical analyses were performed using GraphPad Prism 8 (GraphPad Software Inc., CA, USA) and band quantifications were performed using Bio-Rad Image Lab 5.1 software (Bio-Rad). |

For manuscripts utilizing custom algorithms or software that are central to the research but not yet described in published literature, software must be made available to editors and reviewers. We strongly encourage code deposition in a community repository (e.g. GitHub). See the Nature Portfolio guidelines for submitting code & software for further information.

## Data

Policy information about availability of data

All manuscripts must include a data availability statement. This statement should provide the following information, where applicable:
- Accession codes, unique identifiers, or web links for publicly available datasets
- A description of any restrictions on data availability
- For clinical datasets or third party data, please ensure that the statement adheres to our policy

The authors confirm that the data supporting the findings of this study are available within the article and/or its Supplementary Information. Source data for all

immunoblots are provided with the paper. Raw DNA and RNA sequencing data, as well as neuroimaging data from the individuals herein reported, are securely stored in a controlled-access repository at the UCL Institute of Neurology, University College London, London, UK, and are not publicly available as they comprise part of individuals medical records and contain patient identifiable data. Access to these data is subject to ethical restrictions and will be granted only to clinicians and/or researchers who enter into an appropriate research agreement. These data are also available from the corresponding author upon request subject to completion of any required material transfer agreements (MTAs) and compliance with institutional and legal regulations. Data from the National Genomic Research Library (NGRL) used in this research are available within the secure Genomics England Research Environment. Access to NGRL data is restricted to adhere to consent requirements and protect participant privacy. Data used in this research include: 100,000 Genomes rare disease main release dataset (tiering data). Access to NGRL data is provided to approved researchers who are members of the Genomics England Research Network, subject to institutional access agreements and research project approval under participant-led governance. For more information on data access, visit: https://www.genomicsengland.co.uk/research

## Research involving human participants, their data, or biological material

Policy information about studies with human participants or human data. See also policy information about sex, gender (identity/presentation), and sexual orientation and race, ethnicity and racism.

| | |
|---|---|
| Reporting on sex and gender | Sex information for individual participants is provided in Tables 1 and 2 |
| Reporting on race, ethnicity, or other socially relevant groupings | Participants were recruited through international clinical and research collaborations, and geographic origin is reported where relevant for genetic interpretation |
| Population characteristics | The study population consisted of 13 affected individuals from 12 unrelated families with biallelic variants in COXFA4. Participants presented with a Leigh syndrome spectrum disorder characterised by early-onset neurodevelopmental delay, neurodegeneration, and mitochondrial dysfunction. Clinical, genetic, and biochemical characteristics are detailed in Tables 1 and 2. Age at presentation, clinical features, and molecular findings are reported where available. Functional analyses were performed using patient-derived fibroblasts from a subset of individuals (S1–S5 and S14). |
| Recruitment | Participants were identified through international data-sharing platforms and collaborations, including GeneMatcher, the RD-Connect Genome-Phenome Analysis Platform (GPAP), the 100,000 Genomes Project, the NHS England Genomic Medicine Service, and direct collaboration with national and international clinical and research groups. Recruitment was based on the presence of biallelic COXFA4 variants. |
| Ethics oversight | Written informed consent for genetic testing and data sharing was obtained from all participants or their legal guardians, in accordance with the Declaration of Helsinki via several research studies depending on the individual's country of origin. Specifically, the Medical Research Council (UK) International Centre for Genomic Medicine in Neuromuscular Diseases (ICGNMD) was approved by the relevant Research Ethics Committee (REC) [London—Camberwell St Giles REC (REC ref. 19/LO/1796)]. The 100,000 Genomes Project was approved by the relevant REC [East of England—Cambridge South (REC ref. 14/EE/1112)], and all participants provided informed consent. The study was also approved by the REC at the Institute of Neurology University College London (REC ref, 09/H0716/76). The Department of Clinical Research and Development Public Assistance Hospitals of Paris, in connection with the Ministry of Higher Education and Research located in Paris, France, approved skin fibroblast harvest, research use, and conservation in a research biorepository maintained in Bicêtre Hospital, Paris, France, under declaration DC 2009-939. |

Note that full information on the approval of the study protocol must also be provided in the manuscript.

# Field-specific reporting

Please select the one below that is the best fit for your research. If you are not sure, read the appropriate sections before making your selection.

[x] Life sciences [ ] Behavioural & social sciences [ ] Ecological, evolutionary & environmental sciences

For a reference copy of the document with all sections, see nature.com/documents/nr-reporting-summary-flat.pdf

# Life sciences study design

All studies must disclose on these points even when the disclosure is negative.

| | |
|---|---|
| Sample size | Sample size was determined by the availability of affected individuals with biallelic COXFA4 variants identified through international data-sharing platforms and collaborators. A total of 13 affected individuals from 12 unrelated families were included in the genetic and clinical analyses. Functional experiments were performed on patient-derived fibroblasts where material was available (S1–S5 and S14), with the number of biological replicates specified in the figure legends. 3-4 fibroblasts control lines were used. Sample sizes were sufficient to detect reproducible and statistically significant effects. |
| Data exclusions | No data were excluded from the analyses. All reported ndividuals with biallelic COXFA4 variants and available clinical, genetic, or functional data were included. |
| Replication | Where applicable, experiments were performed using multiple biological replicates, as indicated in the figure legends.Key findings were replicated across multiple independent patient-derived cell lines and experiments where validated using complementary techniques |
| Randomization | Randomization was not applicable to this study. |
| Blinding | Blinding was not applied |

# Reporting for specific materials, systems and methods

We require information from authors about some types of materials, experimental systems and methods used in many studies. Here, indicate whether each material, system or method listed is relevant to your study. If you are not sure if a list item applies to your research, read the appropriate section before selecting a response.

## Materials & experimental systems

| n/a | Involved in the study |
| --- | --- |
| ☐ | ☒ Antibodies |
| ☐ | ☒ Eukaryotic cell lines |
| ☒ | ☐ Palaeontology and archaeology |
| ☒ | ☐ Animals and other organisms |
| ☐ | ☒ Clinical data |
| ☒ | ☐ Dual use research of concern |
| ☒ | ☐ Plants |

## Methods

| n/a | Involved in the study |
| --- | --- |
| ☒ | ☐ ChIP-seq |
| ☒ | ☐ Flow cytometry |
| ☐ | ☒ MRI-based neuroimaging |

## Antibodies

Antibodies used

The following primary antibodies were used in this study: anti-NDUFS3 (Abcam, ab14711; 1:300), anti-MTCO1 (Abcam, ab14705; 1:3000), anti-UQCRC2 (Abcam, ab14745; 1:1000), anti-SDHA (Abcam, ab14715; 1:6000), anti-ATP5A (Abcam, ab14748; 1:9000), anti-COXFA4 (Stratech Scientific, C16821-ABT; 1:1000), anti-COXFA4L2 (Proteintech, 16480-1-AP; 1:500), anti-MTCO2 (Abcam, ab110258), anti-COX6B (Abcam, ab110266), anti-COX5B (Santa Cruz Biotechnology, sc-374417), anti-COXFA4 (Abcam, ab129752), anti-NDUFA9 (Abcam, ab14713), anti-NDUFV1 (Proteintech, 11238-1-AP), anti-COX4 (Abcam, ab62164; ThermoFisher Scientific, MA5-15078), anti-COX6C (Abcam, ab110267), anti-VDAC (Cell Signaling Technology, D73D12; 1:1000), OXPHOS human antibody cocktail (Abcam, ab110411; 1:1000), anti-α-tubulin (Proteintech, 66031-1-Ig; 1:10,000), anti-GAPDH (ThermoFisher Scientific, AM4300; 1:10,000), and anti-β-actin (Cell Signaling Technology, 4970; 1:10,000).
HRP-conjugated secondary antibodies used were anti-rabbit IgG (Dako, P0448; 1:4000) and anti-mouse IgG (Promega, W402B; 1:3000). Fluorescent secondary antibodies used were IRDye 680LT Goat anti-Mouse IgG (Li-Cor Biosciences, 926–68020; 1:10,000) and IRDye 800CW Goat anti-Rabbit IgG (Li-Cor Biosciences, 926–32211; 1:10,000).

Validation

All primary antibodies were used according to the manufacturers' instructions for immunoblotting. Commercial antibodies were validated by the suppliers for human proteins, as stated on the manufacturers' websites. For antibodies recognizing COXFA4 and COXFA4L2, cross-reactivity between the two highly homologous proteins was observed. Band identity was experimentally validated by siRNA-mediated knockdown of COXFA4L2 (Figure 3F; Supplementary Figure 5A), confirming target specificity.

## Eukaryotic cell lines

Policy information about cell lines and Sex and Gender in Research

Cell line source(s)

Primary patient-derived skin fibroblasts were obtained from affected individuals following informed consent and ethical approval. Immortalised fibroblast lines were generated in-house by lentiviral transduction of primary fibroblasts. SH-SY5Y human neuroblastoma cells and H4 human neuroglioma cells were obtained from the American Type Culture Collection (ATCC). HEK293T cells (ATCC) were used for lentiviral packaging. The sex of the primary fibroblast donors is reported in Tables 1 and 2.

Authentication

Commercial cell lines (SH-SY5Y, H4, and HEK293T) were obtained from ATCC. No additional authentication testing was performed by the authors. Primary fibroblasts were derived directly from patient skin biopsies and their sex and was confirmed variant by Sanger sequencing and RNA-seq analysis

Mycoplasma contamination

All cell lines were regularly tested for mycoplasma contamination

Commonly misidentified lines
(See ICLAC register)

N/A

## Clinical data

Policy information about clinical studies

All manuscripts should comply with the ICMJE guidelines for publication of clinical research and a completed CONSORT checklist must be included with all submissions.

Clinical trial registration

N/A

Study protocol

This is not a clinical trial work. Experiments and methods used are fully described in the Methods section of this manuscript.

| | |
|---|---|
| Data collection | Clinical and genetic data were collected through international collaborations, including GeneMatcher, the RD-Connect Genome-Phenome Analysis Platform (GPAP), the 100,000 Genomes Project, the NHS England Genomic Medicine Service, and collaborating clinical centers. Clinical, radiological, biochemical, and molecular data were obtained from medical records, diagnostic testing, and research-based functional studies. |
| Outcomes | N/A |

# Plants

| | |
|---|---|
| Seed stocks | N/A |
| Novel plant genotypes | N/A |
| Authentication | N/A |

# Magnetic resonance imaging

## Experimental design

| | |
|---|---|
| Design type | N/A |
| Design specifications | N/A |
| Behavioral performance measures | N/A |

## Acquisition

| | |
|---|---|
| Imaging type(s) | MRI |
| Field strength | scans were acquired at multiple clinical centers using standard clinical protocols. |
| Sequence & imaging parameters | MRI scans were obtained using standard clinical protocols. |
| Area of acquisition | Whole brain |

Diffusion MRI ☐ Used ☒ Not used

## Preprocessing

| | |
|---|---|
| Preprocessing software | N/A |
| Normalization | N/A |
| Normalization template | N/A |
| Noise and artifact removal | N/A |
| Volume censoring | N/A |

## Statistical modeling & inference

| | |
|---|---|
| Model type and settings | N/A |
| Effect(s) tested | N/A |

Specify type of analysis: ☐ Whole brain ☐ ROI-based ☐ Both

| | |
|---|---|
| Statistic type for inference (See Eklund et al. 2016) | N/A |
| Correction | N/A |

## Models & analysis

| n/a | Involved in the study | |
|---|---|---|
| ☒ | ☐ | Functional and/or effective connectivity |
| ☒ | ☐ | Graph analysis |
| ☒ | ☐ | Multivariate modeling or predictive analysis |
