# Supplementary material for: COXFA4L2 upregulation preserves residual cytochrome c oxidase activity in COXFA4-related Leigh-like encephalopathy
Source: Nat Commun. 2026 May 30;17:7026. doi: 10.1038/s41467-026-73455-9 (PMC13392013; doi:10.1038/s41467-026-73455-9)
Supplement: Supplementary file 1 — Supplementary information [file 41467_2026_73455_MOESM1_ESM.pdf]

COXFA4L2 upregulation preserves residual cytochrome *c* oxidase activity in *COXFA4*-related Leigh-like encephalopathy

Supplementary Results

Clinical presentations of individuals with biallelic *COXFA4* variants

Subject 1 (S1) was a 29-year-old female of Sri Lankan descent born to non-consanguineous parents. At birth, she presented with hypotonia, poor feeding, and lethargy. Both motor and language milestones were delayed: she achieved independent sitting at 18 months, walking at 2 years, and spoke her first words at 4 years. During childhood, she developed progressive proximal muscle weakness and developmental regression, leading to loss of ambulation at 13 years of age. Neurological examination at age 20 years included spasticity, cerebellar ataxia, dysarthria, limb dystonia, and a mild tremor of the head and arms. There was moderate intellectual disability, requiring assistance with all activities of daily living. Additional clinical features included obesity (BMI 40), obstructive sleep apnoea requiring nocturnal CPAP, type 2 diabetes, and cardiac abnormalities, including Wolff–Parkinson–White (WPW) syndrome and mild left ventricular hypertrophy at 15 years. Brain MRI at the age of 25 years demonstrated thinning of the corpus callosum, cerebral atrophy, and periventricular and temporal white matter involvement (Figure 2). Plasma lactate was raised (3.33 mmol/L; reference range: 0.5-2.2), consistent with a mitochondrial respiratory chain defect. Muscle tissue revealed reduced complex IV activity, expressed as a ratio to citrate synthase activity (0.009; reference range: 0.03-0.14). Complex I activity was found to be within normal limits (0.162; reference range: 0.104-0.268).

Subject 2 (S2) was a 3-year-old female of Sri Lankan descent born to non-consanguineous parents from the same geographic region as S1. She presented with respiratory distress and elevated blood lactate levels at birth. Clinical features include hypotonia and delayed motor and language development. Additional clinical findings included bilateral cataracts, vomiting, and feeding difficulties. Cardiac evaluation showed biventricular hypertrophy. Brain MRI at 6 days of life showed small bilateral lesions in the posterior parietal periventricular white matter. Patient-derived fibroblasts showed decreased complex IV activity (0.419×10^{-3} K·sec^{-1}·unit citrate synthase^{-1}; controls, n=8: $1.083 \pm 0.186 \times 10^{-3}$)

Subject 3 (S3) was a 3-year-old female of Sri Lankan origin born to non-consanguineous parents. At birth, she presented with respiratory distress and failure to thrive. During infancy, she developed muscle weakness, ptosis, and cerebellar ataxia. Motor milestones were delayed, with walking at 24 months; however, other developmental milestones were met appropriately. Brain MRI at 24 months revealed mild cerebral atrophy and symmetrical white matter signal abnormalities in the supra- and subtentorial regions, including the putamen and brainstem. Plasma lactate (6.2 mmol/L; reference range: 0.5 – 2.2 mmol/L) and lactate:pyruvate (28.2; reference range: 0 – 18) were increased.

Subject 4 (S4) was a 9-year-old male of Afghan descent born at 32 weeks of gestation to consanguineous (second cousin) parents. At birth, he presented with respiratory distress and feeding difficulties requiring nasogastric tube feeding. He later developed lower limb spasticity, cerebellar ataxia, dysarthria, and generalised tonic-clonic seizures from 2 years and 10 months. He had severe intellectual disability and significant global developmental delay, necessitating continuous assistance. Brain MRI at 16 months showed multifocal symmetrical white matter abnormalities involving the corpus callosum and dentate nuclei. Plasma lactate was elevated (6-12 mmol/L; normal range 0.5-2.2 mmol/L) whilst complex IV activities were decreased in both muscle tissue (0.164×10^{-3} $K \cdot sec^{-1} \cdot$unit citrate synthase^{-1}; controls, n=25: $1.124 \pm 0.511 \times 10^{-3}$) and fibroblasts ($0.118 \times 10^{-3}$ $K \cdot sec^{-1} \cdot$unit citrate synthase^{-1}; controls, n=8: $1.083 \pm 0.186 \times 10^{-3}$).

Subject 5 (S5) was a 17-year-old male of Turkish descent, born to consanguineous (first cousin) parents. He presented with neonatal respiratory distress and feeding difficulties, requiring an 8-day admission to the neonatal intensive care unit. He subsequently developed lower limb spasticity and pyramidal signs, including brisk reflexes, clonus, and extensor plantar responses. He had severe intellectual disability and was non-ambulatory. Additional clinical features include facial and bulbar myopathy, lumbar scoliosis, short stature, and a high-arched palate. Cardiac evaluation revealed concentric left ventricular hypertrophy. Plasma lactate was elevated (36.6 mg/dl; reference range 4.5-19.8 mg/dl).

Subject 14 (S14) was a 45-year-old male of Pakistani descent from a consanguineous family. He presented with lactic acidosis during the neonatal period and later developed spastic diplegia. His clinical features have been previously described[18] (23746447, subject III-4) and

include moderate intellectual disability with delayed speech, sensory axonal neuropathy, dysphagia, generalised tonic–clonic and myoclonic seizures (from age 13) and progressive spasticity with pyramidal signs. Additional features include primary hyperparathyroidism, short stature, and constipation. Brain MRI was normal at age 17 years but repeat imaging at the age of 25 years demonstrated T2-weighted hyperintensities in the deep cerebral white matter. Muscle tissue confirmed reduced complex IV activity (0.008; reference range: 0.014–0.034).

Supplementary Figure 1

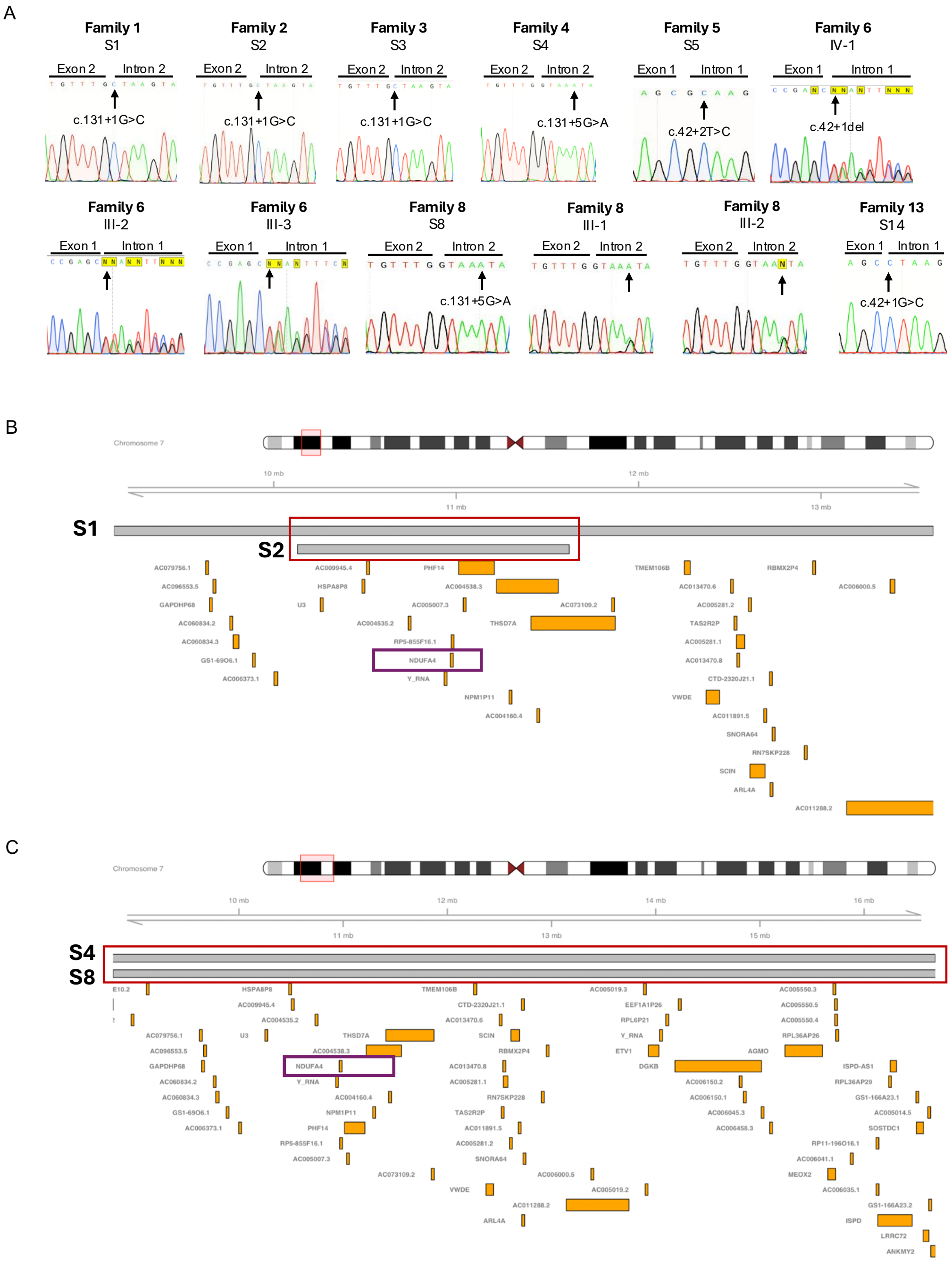

Supplementary Figure 1. Details of *COXFA4* variants (**A**) Chromatograms from Sanger sequencing of *COXFA4* (RefSeq: NM_002489.4) genotypes for probands, parents, and siblings of families 1-13. (**B**) S1 and S2 haplotype analysis. Both S1 and S2 (c.131+1G>C) share a haplotype and are homozygous in the region [hg38] chr7: 10,129,376-11,619,685 (red box). The regions of homozygosity are 4.5 kb and 1.5 kb, respectively. (**C**) S4 and S8 haplotype analysis. Both S4 and S8 (c.131+5G>A) share a haplotype and are homozygous in the region [hg38] chr7: 8,792,148- 16,683,217 (red box). The sizes of the regions of homozygosity of S4 and S8 are 8 kb and 8.9 kb, respectively.

Supplementary Figure 2

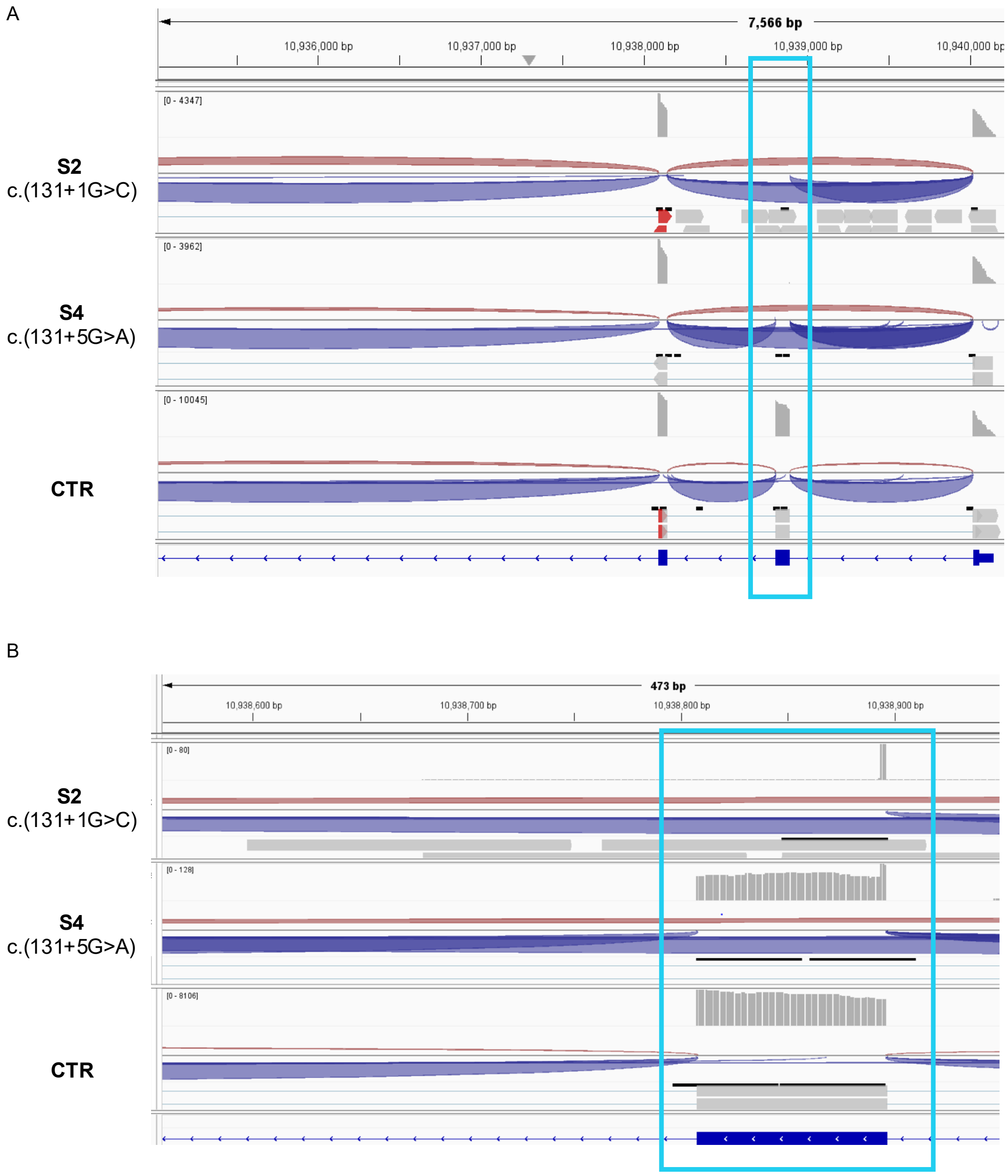

Supplementary Figure 2. Aberrant *COXFA4* splicing in patient-derived fibroblasts (S2 and S4) and healthy control (CTR). (A) Integrative Genomics Viewer (IGV) screenshot showing the genomic region spanning the *COXFA4* locus on the reverse strand. Coverage is shown in grey bars. Affected samples show markedly reduced usage of canonical splice junctions and activation of cryptic splice sites, resulting in exon skipping (cyan box). (**B**) Higher magnification of the boxed region from (A) showing normal coverage of the exon in the control, which is reduced in S4 and absent in S2. This is reflective of exon skipping. The single column in S2 is a mismapping artefact.

Supplementary Figure 3

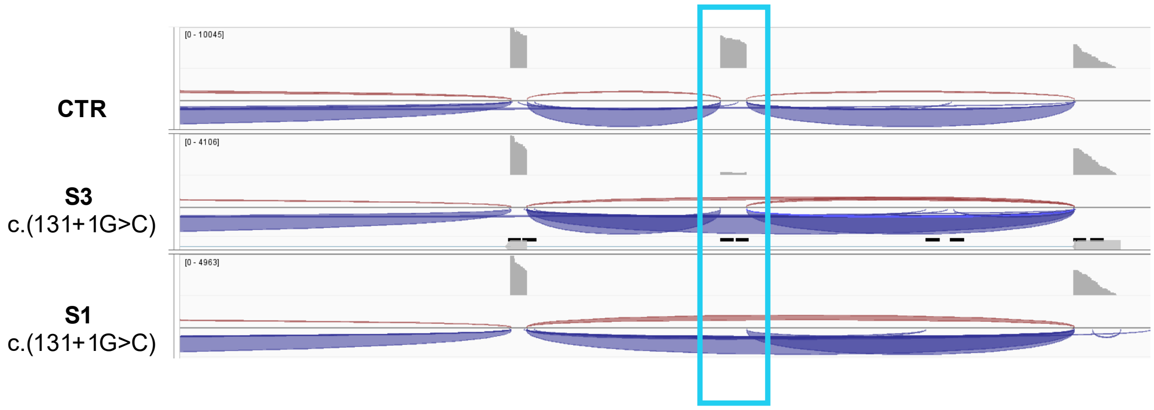

Supplementary Figure 3. Aberrant *COXFA4* splicing in patient-derived fibroblasts (S1 and S3) and healthy control (CTR). Integrative Genomics Viewer (IGV) screenshots of RNA-seq data from affected individuals (S1 and S3) showing exon skipping and the usage of cryptic splice sites (cyan box). Read coverage (grey bars), shows that the exon is nearly absent in S3 and absent in S1.

Supplementary Figure 4

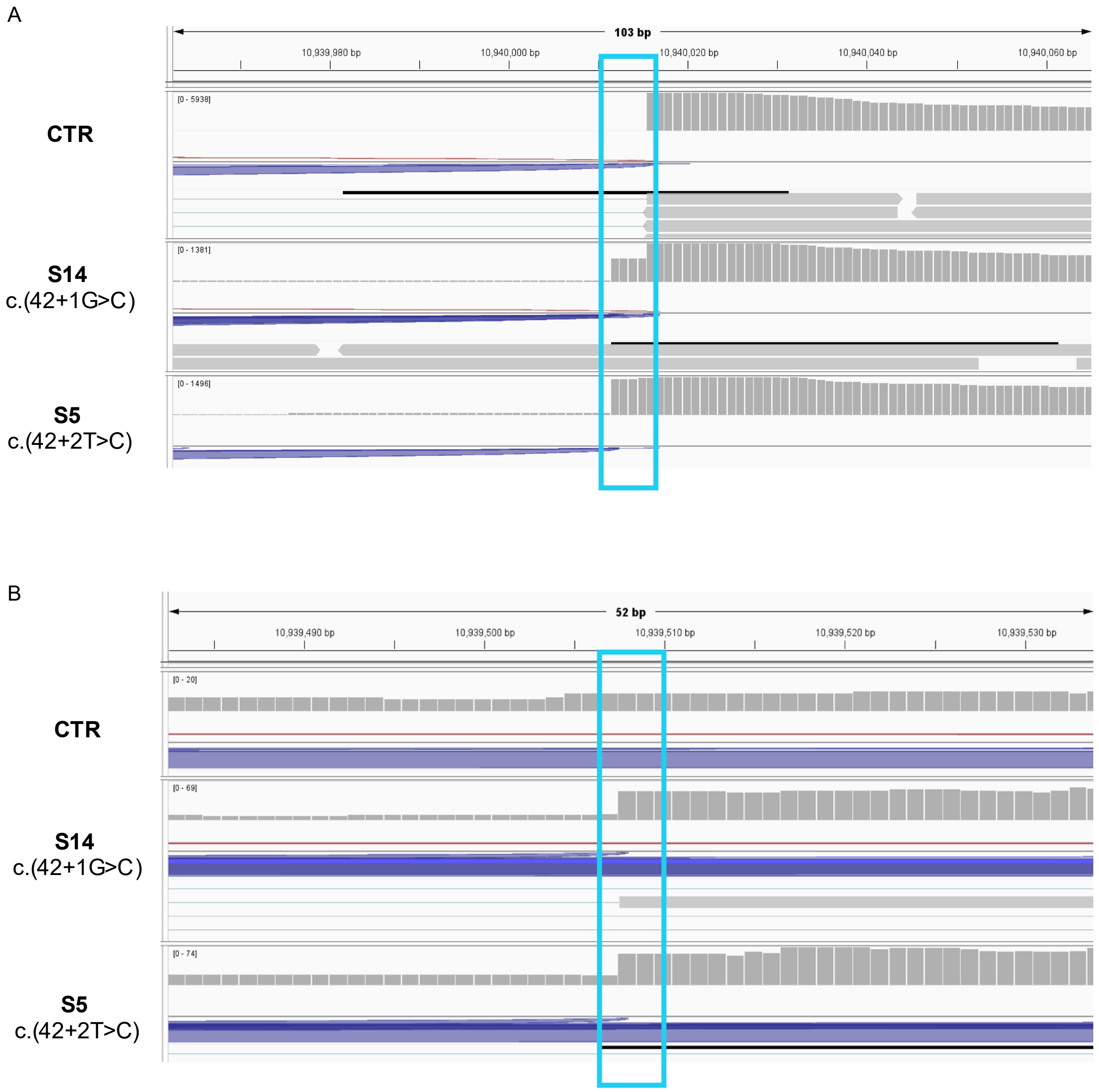

Supplementary Figure 4. Aberrant *COXFA4* splicing in patient-derived fibroblasts (S14 and S5) and healthy control (CTR). (**A**) Integrative Genomics Viewer (IGV) screenshot of RNA-seq data showing the genomic region spanning the *COXFA4* locus. Coverage is shown as grey bars. Affected samples show that exon 1 ends at position 10,940,012 with activation of a novel donor site (cyan box). (**B**) IGV screenshot of RNA-seq data showing a possible additional donor gain event at position 10,939,508.

Supplementary Figure 5

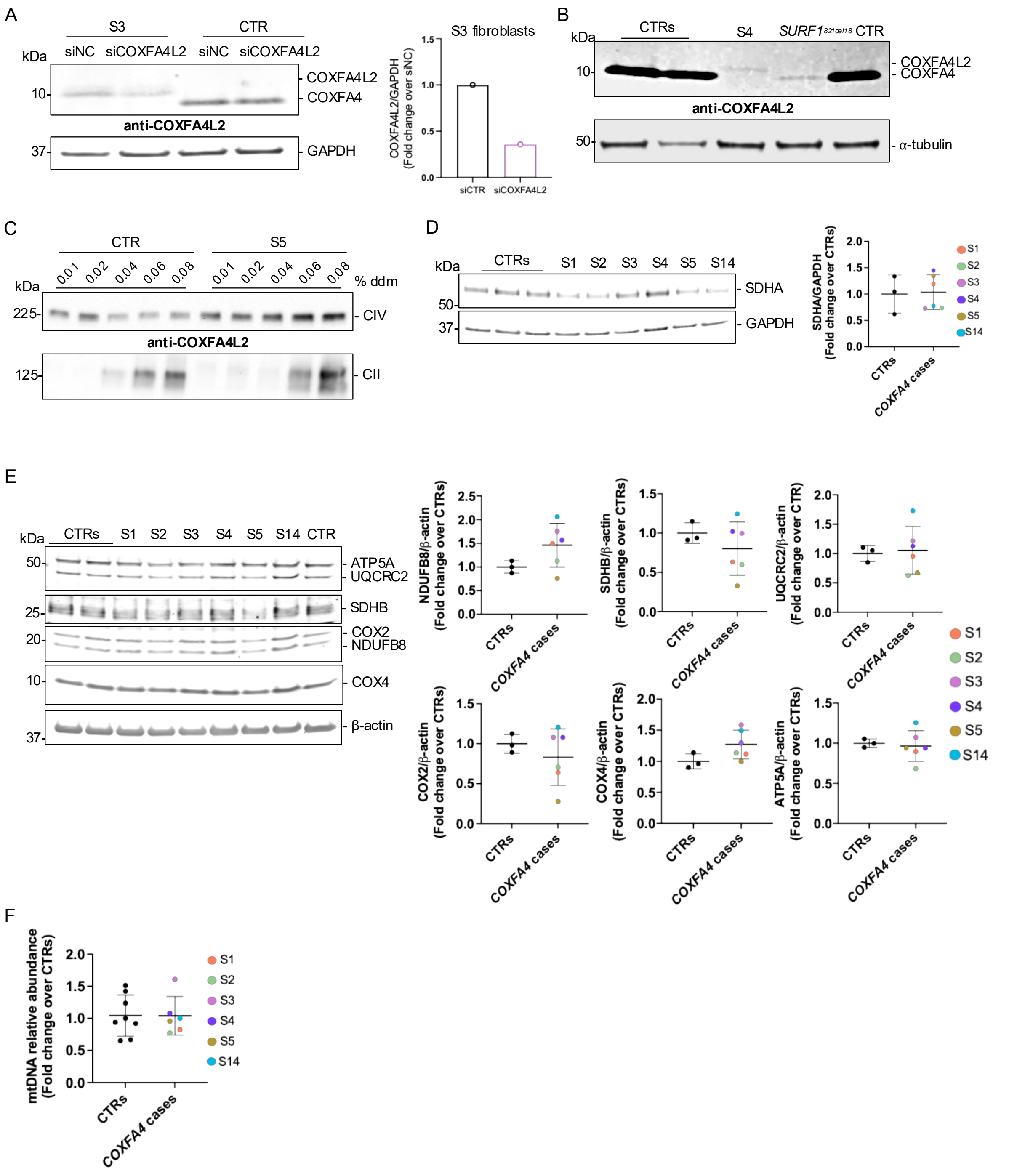

Supplementary Figure 5. Characterisation of *COXFA4* patient-derived primary fibroblasts. (**A**) Immunoblot analysis of COXFA4 and COXFA4L2 expression in primary fibroblasts from a healthy control (CTR) and patient S3 following siRNA-mediated knockdown (KD) of COXFA4L2 (siCOXFA4L2) or non-targeting control siRNA (siNC). GAPDH was used as a loading control. (**B**) Immunoblot analysis of COXFA4 and COXFA4L2

expression in primary fibroblasts from a healthy control (CTR), *COXFA4* patient (S4) and patient carrying a pathogenic variant in the *SURF1* gene (c.821del18). The higher molecular weight band corresponding to COXFA4L2 was present only in S4. α-tubulin was used as a loading control. (**C**) Immunoblot analysis of blue-native polyacrylamide gels loaded with total cell lysate extracted from primary fibroblasts from a healthy control (CTR) and patient S5. Proteins were extracted using the following concentrations of n-dodecyl β-D-maltoside (DDM): 0.01%; 0.02%; 0.04%; 0.06%; 0.08%. Proteins were run on 8%–16% polyacrylamide gradient blue-native gels and probed for COXFA4L2 to assess the COX associations. (**D**) SDS-PAGE analysis of SDHA protein steady-state levels in total cellular protein extracts from three healthy controls (CTRs) and six *COXFA4* patients (S1–S5, S14). GAPDH was used as a loading control. (**E**) Immunoblot detecting the expression of OXPHOS subunits NDUFB8 (complex I), SDHB (complex II), UQCRC2 (complex III_2), COX2 and COX4 (complex IV) and ATP5A (complex V) in primary fibroblasts from four healthy controls (CTRs), and six *COXFA4* patients (S1–S5, S14). β-actin was used as a loading control. (**F**) Mitochondrial DNA (mtDNA) abundance in primary fibroblasts from eight healthy controls (CTRs) and six *COXFA4* patients (S1–S5, S14). Error bars indicate standard deviation (SD), n=3-8, where each data point represents an independent biological sample.

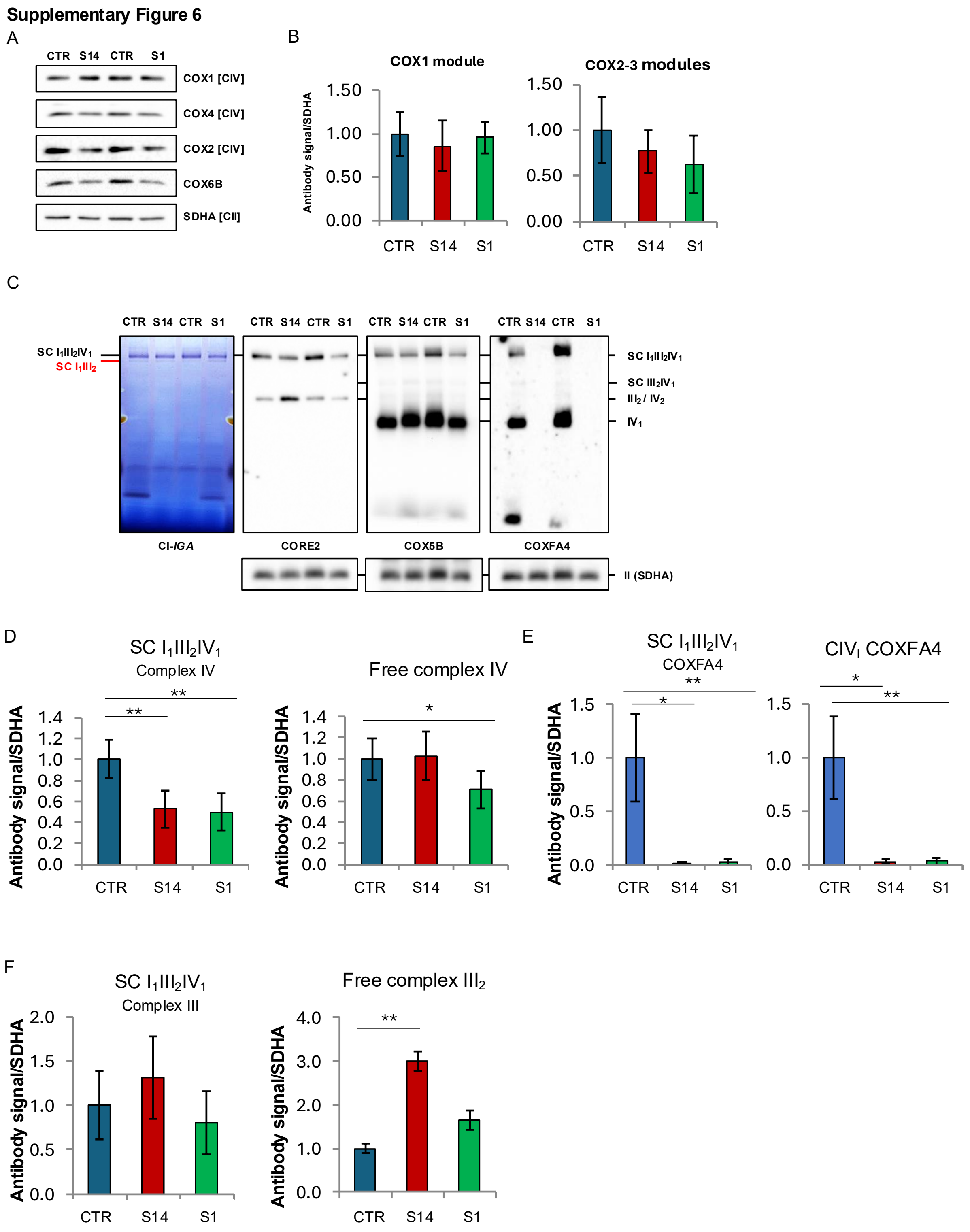

Supplementary Figure 6. Assembly state of OXPHOS complexes and supercomplexes in immortalised *COXFA4* patient-derived fibroblasts. **(A)** Representative immunoblots of steady-state COX subunits in two controls (CTR) and COXFA4-deficient fibroblasts (S1 and

S14), showing COX1 module (COX1, COX4), COX2 module (COX2) and COX3 module (COX6B). SDHA was used as loading control. (**B**) Quantification of COX modules shown in (A). Signals were normalised to SDHA and expressed relative to CTR. (**C**) BN-PAGE of digitonin-solubilised mitochondria. Left panel, complex I in-gel activity (CI-I*GA*) with supercomplex positions indicated. Middle and right panel, BN-blots probed for CORE2 (complex III_2), COX5B (complex IV) and COXFA4. COXFA4 was detected using the Abcam antibody (ab129752). SDHA was used as loading control. (**D–F**) Densitometry of BN-blots: (**D**) COX5B in respirasome ($I_1III_2IV_1$) and free complex IV; (**E**) COXFA4 in respirasome ($I_1III_2IV_1$) and free complex IV; (**F**) CORE2 in SC $I_1III_2IV_1$ and free complex III_2. All values normalised to SDHA and expressed relative to CTR. Data are presented as mean ± SD. Statistical comparisons between CTR, S1 and S14 were performed using one-way ANOVA with Tukey's multiple comparisons test: *$p<0.05$; **$p<0.01$.

Supplementary Figure 7

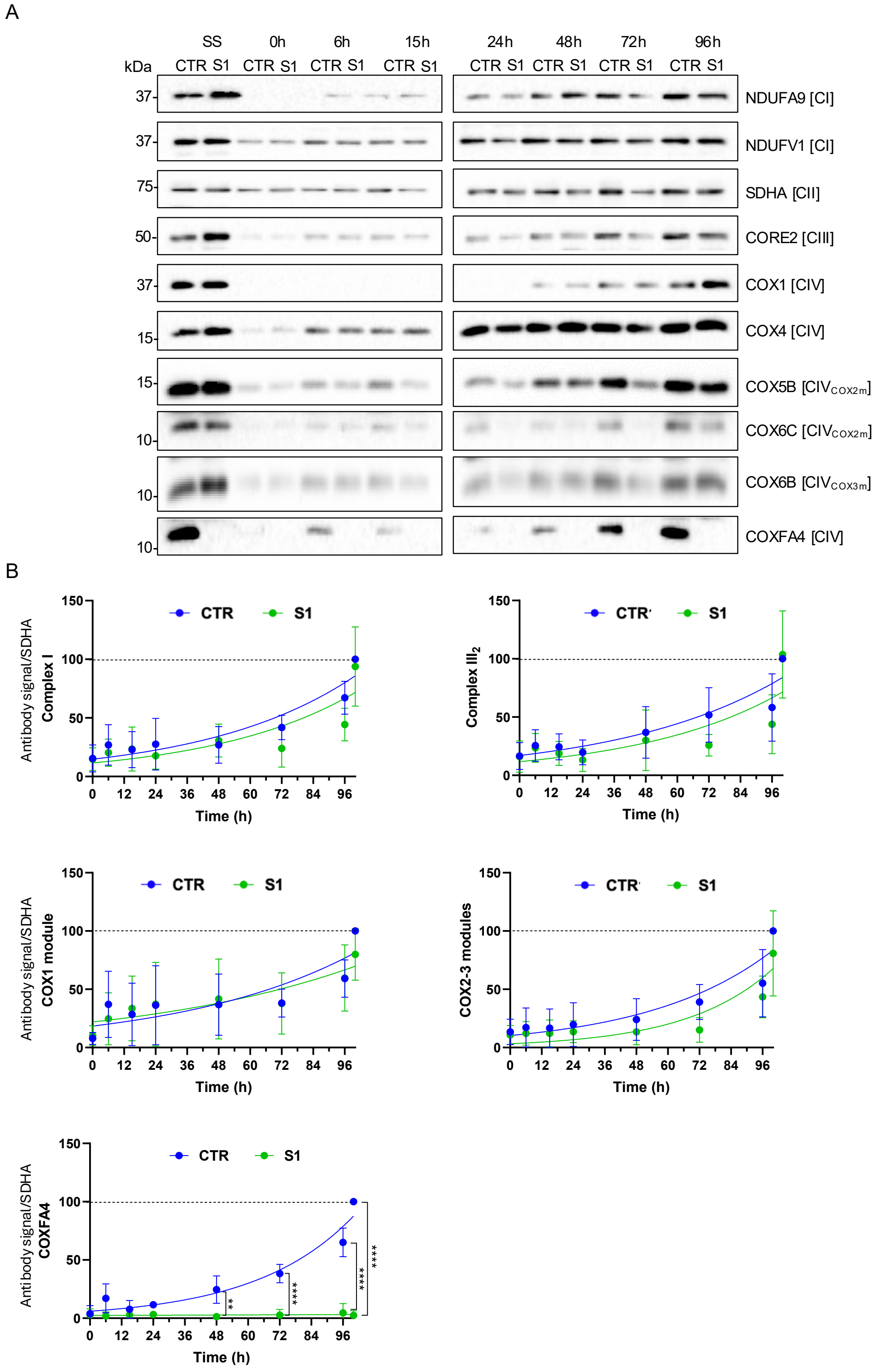

Supplementary Figure 7. SDS–PAGE-based analysis of the reappearance of individual mitochondrial respiratory chain subunits following reversible inhibition of mitochondrial translation in immortalised COXFA4 patient-derived fibroblasts. (A) Representative immunoblot of mitochondrial protein from control (CTR) and *COXFA4* patient-derived immortalised fibroblasts (S1) cultured for 6 days in the presence of 15µg/ml doxycycline and

collected at different time points (0, 6, 15, 24, 48, 72 and 96 h) following doxycycline removal. (**B**) Quantification of incorporation rates of mitochondrial respiratory chain complex subunits: NDUFA9 and NDUFV1 (complex I); CORE2 (complex III_2); COX1 and COX4 (COX1 module); COX5B, COX6B, COX6B (COX2–COX3 modules); and COXFA4. COXFA4 was detected using the Abcam antibody (ab129752). Signal intensities from three independent experiments were quantified by densitometry, normalised to SDHA, and expressed relative to steady-state (SS) levels, set to 100% (dotted line). Normalised values were fitted to an exponential recovery curve. Data are presented as mean ± SEM. Statistical comparisons between CTR and S1 were performed using two-way ANOVA with Tukey's multiple comparisons test: ** $p<0.01$; **** $p<0.001$.

Supplementary Figure 8

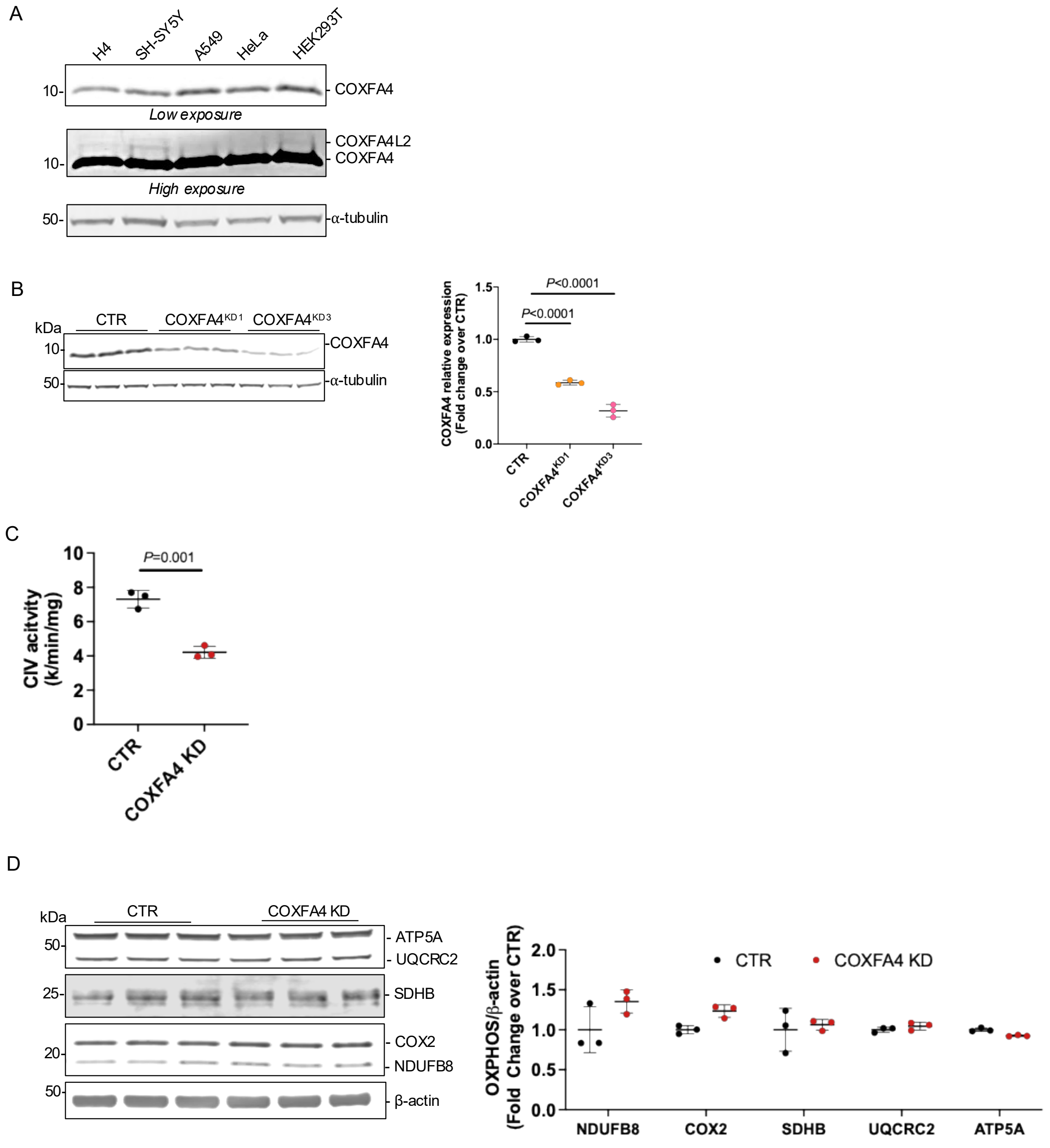

Supplementary Figure 8. Analysis of the expression and functional consequences of COXFA4 knockdown. (**A**) Immunoblot analysis of H4, SH-SY5Y, A549, HeLa and HEK293T showing the expression levels of COXFA4 and COXFA4L2. Membranes were probed with a COXFA4L2 antibody (Proteintech, 16480-1-AP), which also detects COXFA4. α-tubulin was used as a loading control. (**B**) Immunoblot analysis of H4 cells stably transfected with two distinct shRNA constructs targeting COXFA4, showing efficient knockdown of COXFA4 expression. (**C**) Spectrophotometric assay of complex IV enzymatic activity in mitochondrial protein pellets from COXFA4 knockdown (COXFA4 KD). Error bars indicate

standard deviation (SD), n=3, where each data point represents a technical replicate. Statistical significance was determined by paired *t*-test compared to control (CTR). (**D**) Immunoblot detecting the OXPHOS subunits expression NDUFB8 (complex I), SDHB (complex II), UQCRC2 (complex III_2), COX2 (complex IV) and ATP5A (complex V) in SH-SY5Y cells with COXFA4 knockdown (COXFA4 KD). β-actin was used as a loading control. Error bars indicate standard deviation (SD), n=3, where each data point represents an independent biological sample. Statistical significance was determined by unpaired *t*-test compared to control (CTR).

Supplementary Table 1. Residues at the monomer–monomer interface interacting with COXFA4 or COXFA4L2. Residues at the monomer–monomer interface interacting with COXFA4 or COXFA4L2. Residues from different chains of complex IV interacting with either COXFA4 or COXFA4L2 subunits are highlighted in yellow; those unique to COXFA4L2 are highlighted in blue. The first letter denotes the amino acid residue, whereas the second letter denotes the protein chain, according to the chain letters reported in the crystallised complex IV (according to 5z62.pdb).

COXFA4	COX1	COX2	COX3	COX5B	COX6B1	COXFA4L2
IN5	QA178	DB57	LC106	DF96	IH9	RX9
QN8	YA179	QB59	AC107	TF98	PH16	FX10
KN11	MA271	EB62	PC108		DH18	QX13
HN12	WA275	TB63	HC115		SH19	IX14
PN13	AA276	TB66	WC116		RH20	RX16
SN14	MA278	IB67	WC259		FH21	HX17
LN15	SA279	AB70			PH22	PX18
PN17	FA282	RB82			NH23	IX20
LN18	LA283	YB85			NH25	PX22
FN19	IA286	MB86			RH28	MX23
FN21	DA298	DB88			WH31	IX24
IN22	VA299	EB89			QH32	LX26
TN24	DA300	VB90			DH36	IX27
AN26	TA301	DB92			RH39	MX31
AN29	YA304				CH54	SX33
YN32	IA311				WH56	AX34
LN33	IA314				YH57	YX37
RN35	PA315					LX38
LN36	VA318					RX40
NN40						LX41
PN41						PX46
DN42						DX47
VN43						VX48
CN44						CX49
WN45						WX50
NN48						KX53
EN51						NX54
PN52						EX57
KN55						PX58
LN56						RX61
PN58						LX62
NN59						PX64
DN60						NX65
YN62						DX66
FN64						YX68
YN65						FX70
SN66						LX71
VN67						AX72
NN68						VX73
DN70						SX74
YN71						DX76
KN73						YX77
EN77						KX79
PN79						KX81
DN80						DX83
						PX85
						DX86

Supplementary Table 2. Free energy of interaction at the interface between cytochrome *c* oxidase subunits and COXFA4 or COXFA4L2 in the investigated models. Free energy of interaction at the interface between cytochrome c oxidase subunits and COXFA4 or COXFA4L2 in the investigated models. Interaction energies (in kcal/mol) were calculated using the FoldX AnalyseComplex tool. More negative values indicate stronger binding and higher affinity at the protein–protein interface of the indicated complexes. Energy terms and their individual contributions are reported in accordance with FoldX conventions. The first column lists the energy components, while columns 2–3 display the corresponding values for each protein complex (including protein name and chain ID). All numerical values, including units and decimal precision, are reported as directly output by the FoldX software. For definitions of the energy terms and additional methodological details, see: http://foldxsuite.crg.eu/command/AnalyseComplex

Group1	COXFA4 (chain N)	COXF4L2 (chain X)
Group2	ABCDEFGHIJKLM	ABCDEFGHIJKLM
IntraclashesGroup1	12.7704	40.1545
IntraclashesGroup2	141.081	144.114
**Interaction Energy**	**-20.1211**	**-24.1923**
Backbone Hbond	-2.15181	-1.96906
Sidechain Hbond	-11.5143	-7.44866
Van der Waals	-30.222	-30.5842
Electrostatics	-2.68336	-2.72354
Solvation Polar	35.3095	34.7279
Solvation Hydrophobic	-42.492	-43.2695
Van der Waals clashes	7.07293	5.61238
entropy sidechain	18.3856	17.7253
entropy mainchain	8.73959	7.96555
torsional clash	0.219999	0.405708
backbone clash	7.11084	6.54332
helix dipole	0.162083	0.286167
electrostatic kon	-0.960372	-0.951781
energy Ionisation	0.0130622	0.0314611
Entropy Complex	2.384	2.384
Number of Residues	1860	1861
Interface Residues	100	106

Supplementary Table 3. Nuclear-encoded subunits of complex IV associated with human disease

Gene	Variant	Number of cases	Clinical phenotype	**References**
COXFA4	c.42+1G>C	3	Leigh-like syndrome with dystonia, ataxia and lactic acidosis	1
	10,969,473_10,982,428 del	1	Leigh-like syndrome with dystonia, ataxia and lactic acidosis	2
	10,972,975_10,981,04 del	3	Leigh-like syndrome with dystonia, ataxia and lactic acidosis	3
	c.131+1G>C	4	Leigh-like syndrome with dystonia, ataxia and lactic acidosis	Present study
	c.131+5G>A	4	Leigh-like syndrome with dystonia, ataxia and lactic acidosis	
	c.42+2T>C	1	Leigh-like syndrome with dystonia, ataxia and lactic acidosis	
	c.42+1del	1	Leigh-like syndrome with dystonia, ataxia and lactic acidosis	
	c.43-1G>A	1	Leigh-like syndrome with dystonia, ataxia and lactic acidosis	
	10,932,820_10,940,833 del	2	Leigh-like syndrome with dystonia, ataxia and lactic acidosis	
	10,890,006_10,953,862 del	3	Leigh-like syndrome with dystonia, ataxia and lactic acidosis	
COX4I1	c.303_304delinsTT	1	Short stature, dysmorphic features, Fanconi anaemia	4, 5
	c.454C>A	3	Leigh-like syndrome with encephalopathy, developmental regression, intellectual disability, seizures, hypotonia	6, 7
COX4I2	c.412G>A	4	Congenital exocrine pancreatic insufficiency, dyserythropoietic anemia, calvarial hyperostosis	8
COX5A	c.266T>G	1	Failure to thrive, lactic acidosis, hypoglycemia, short stature	9
	c.319C>T	2	Pulmonary arterial hypertension, lactic acidemia and failure to thrive	10
COX6A1	c.247-7_247-3del	3	Charcot-Marie-Tooth disease	11
	c.247-7_247-3del	1	Charcot-Marie-Tooth disease	12
COX6A2	c.117C>A	1	Myopathy, cardiomyopathy	13
	c.117C>A, c.127T>C	1	Myopathy	13
COX6B1	c.221G>A	2	Mitochondrial encephalomyopathy	14
	c.58C>T	1	Encephalomyopathy, hydrocephalus, hypertrophic cardiomyopathy	15
COX7B	c.196delC	1	Microphthalmia with linear skin lesions	16
	c.41-2A>G	1		
	c.55C>T	2		
COX8A	c.115-1G>C	1	Leigh-like syndrome, leukodystrophy, severe epilepsy	17

References

1. Pitceathly, R. D. S. *et al.* NDUFA4 mutations underlie dysfunction of a cytochrome c oxidase subunit linked to human neurological disease. *Cell Rep* **3**, 1795–1805 (2013).
2. Misceo, D. *et al.* Biallelic NDUFA4 Deletion Causes Mitochondrial Complex IV Deficiency in a Patient with Leigh Syndrome. *Genes (Basel)* **15**, 500 (2024).
3. Marquez, J. *et al.* Polyamine metabolism is dysregulated in COXFA4-related mitochondrial disease. *HGG Adv* **6**, (2025).
4. Abu-Libdeh, B. *et al.* Mutation in the COX4I1 gene is associated with short stature, poor weight gain and increased chromosomal breaks, simulating Fanconi anemia. *Eur J Hum Genet* **25**, 1142–11146 (2017).
5. Douiev, L. *et al.* Replicative Stress Coincides with Impaired Nuclear DNA Damage Response in COX4-1 Deficiency. *Int J Mol Sci* **23**, (2022).
6. Pillai, N. R. *et al.* Biallelic variants in COX4I1 associated with a novel phenotype resembling Leigh syndrome with developmental regression, intellectual disability, and seizures. *Am J Med Genet A* **179**, 2138–2143 (2019).
7. Liu, Z. *et al.* Compound heterozygosity of a De novo 16q24.1 deletion and missense mutation in COX4I1 leads to developmental regression, intellectual disability, and seizures. *Epilepsia Open* **10**, 942–947 (2025).
8. Shteyer, E. *et al.* Exocrine pancreatic insufficiency, dyserythropoeitic anemia, and calvarial hyperostosis are caused by a mutation in the COX4I2 gene. *Am J Hum Genet* **84**, 412–417 (2009).
9. Torraco, A. *et al.* A novel homozygous variant in COX5A causes an attenuated phenotype with failure to thrive, lactic acidosis, hypoglycemia, and short stature. *Clin Genet* **102**, 56–60 (2022).
10. Baertling, F. *et al.* Mutation in mitochondrial complex IV subunit COX5A causes pulmonary arterial hypertension, lactic acidemia, and failure to thrive. *Hum Mutat* **38**, 692–703 (2017).
11. Tamiy, G. *et al.* A mutation of COX6A1 causes a recessive axonal or mixed form of Charcot-Marie-Tooth disease. *Am J Hum Genet* **95**, 294–300 (2014).
12. Laššuthová, P., Beharka, R., Krůtová, M., Neupauerová, J. & Seeman, P. COX6A1 mutation causes axonal hereditary motor and sensory neuropathy - the confirmation of the primary report. *Clin Genet* **89**, 512–514 (2016).
13. Inoue, M. *et al.* COX6A2 variants cause a muscle-specific cytochrome c oxidase deficiency. *Ann Neurol* **86**, 193–202 (2019).
14. Massa, V. *et al.* Severe infantile encephalomyopathy caused by a mutation in COX6B1, a nucleus-encoded subunit of cytochrome c oxidase. *Am J Hum Genet* **82**, 1281–1289 (2008).
15. Abdulhag, U. N. *et al.* Mitochondrial complex IV deficiency, caused by mutated COX6B1, is associated with encephalomyopathy, hydrocephalus and cardiomyopathy. *Eur J Hum Genet* **23**, 159–164 (2015).
16. Indrieri, A. *et al.* Mutations in COX7B cause microphthalmia with linear skin lesions, an unconventional mitochondrial disease. *Am J Hum Genet* **91**, 942–949 (2012).
17. Hallmann, K. *et al.* Loss of the smallest subunit of cytochrome c oxidase, COX8A, causes Leigh-like syndrome and epilepsy. *Brain* **139**, 338–345 (2016).
