# Supplementary material for: COXFA4L2 upregulation preserves residual cytochrome c oxidase activity in COXFA4-related Leigh-like encephalopathy
Source: Nat Commun. 2026 May 30;17:7026. doi: 10.1038/s41467-026-73455-9 (PMC13392013; doi:10.1038/s41467-026-73455-9)
Supplement: Supplementary file 3 — Peer Review file [file 41467_2026_73455_MOESM3_ESM.pdf]

nature portfolio

Peer Review File

COXFA4L2 upregulation preserves residual cytochrome c oxidase activity in COXFA4-related Leigh-like encephalopathy

Corresponding Author: Dr Micol Falabella

This file contains all reviewer reports in order by version, followed by all author rebuttals in order by version.

Version 0:

Reviewer comments:

Reviewer #1

(Remarks to the Author)
This manuscript reports a large cohort of patients with a Leigh-like phenotype (neurodegenerative disorder) all of whom have biallelic pathogenic variants in COXFA4, a subunit of Complex IV (cytochrome c oxidase). The mutations in the gene are all loss of function, and the protein is undetectable by immunoblot analysis, suggesting that all patients are functionally null for the protein. The loss of COXFA4 results in the upregulation of a paralogue COXFA4L2, which partially compensates for the loss of complex IV activity analyzed in patient fibroblasts. The genetic analysis is completely convincing, and the authors do a rather thorough job of downstream biochemical analysis. This represents an important addition to our understanding of primary mitochondrial disease, and in particular those associated with impaired complex IV activity

I have a few comments/queries that I think should be addressed to improve the manuscript.

(1) In some experiments primary patient fibroblasts are used in others immortalized fibroblasts. There are no descriptions of culture conditions or the agent(s) used to immortalize the cells. Do the immortalized cells remain diploid?
(2) There seems to be a lot of confusion about the specificity of the antibodies used to detect COXFA4 and its paralogue. In the methods section it is stated that the anti-COXFA4 recognizes both proteins. Yet on pg 22 I read the following "This is consistent with the high specificity of the COXFA4 antibody, which does not react with its paralog". Clearly both statements cannot be true, and the data in Fig. 2D show that the antibody detects both. Further, in Figs. 2E,F it is stated that an antibody specific for COXFA4L2 was used but the data shown suggest otherwise. Looks to me that it recognizes both proteins.
(3) I am also puzzled why the antibody directed against the paralog was used in Fig 2.G. COXFA4L2 is barely detectable in control cells, and that is not consistent with detection of the native complex at relatively high levels. Seems to me that they should have used an antibody that is known to recognize complex IV on native gels, otherwise I think the results in this figure are not readily interpretable. Why for instance is the amount of the complex similar in controls and patients. There should be considerably of the fully assembled complex in the patient. I would also like to be convinced that the antibody can recognize the native complex.
(4) I think it is difficult to conclude that the lack of COXFA4 in and of itself impedes respirasome assembly. If there is less of the "free" complex IV, seems to me there will be less in the respirasome. That doesn't necessarily imply that the subunit is somehow necessary for higher order complexes. Also, in Fig 4. what accounts for the very low levels of complex II? It does not have mtDNA-encoded subunits so should not be affected by doxycycline. Further, the data presented in Fig. 4 seem at odds with the data presented in Supp. Fig 8, and I wonder why the antibody against the paralog was not used in the latter experiment. Maybe I am missing something, but I don't understand why low concentrations of DDM do not solubilize the complex from the patient.
(5) I do not get Supp Fig 6 C. On the blot is says the COXFA4L2 antibody was used. In the legend it says the COXFA4 antibody was used. Further, these results do not accord with those in Fig 4 where the complex was seen at low DDM concentrations in controls but not in the patient. Explanation?
(6) I do not think it is possible to conclude anything definitive about the order of assembly of the subunits in complex IV from the data presented in this manuscript.
(7) Very minor -I would settle on immunoblot or Western blot. Personally, I prefer the former.

Reviewer #2

(Remarks to the Author)
This manuscript by Falabella and colleagues presents an extensive investigation that significantly advances our understanding of COXFA4-related mitochondrial disease. The authors describe 13 affected individuals from 12 unrelated families carrying biallelic pathogenic variants in COXFA4. Using a combination of genomic, transcriptomic, biochemical, and structural approaches, they demonstrate that loss of COXFA4 protein leads to a marked reduction in complex IV activity, yet residual enzymatic function is preserved through upregulation of COXFA4L2, a paralog with minimal basal expression. Functional studies in patient-derived fibroblasts and neuronal cells, combined with complementation and knockdown assays, support a model in which COXFA4L2 partially substitutes for COXFA4, maintaining limited COX activity and respiratory capacity.
The study is interesting, well written and the experiments elegant. This work will thus likely have considerable impact in the mitochondrial disease field. However, several aspects require clarification.

The genetic and clinical characterization of the cohort is extremely accurate and consistent with a Leigh-like phenotype. The identification of seven novel variants, including multiple splice-site mutations, adds valuable information to the existing COXFA4 mutational spectrum. However, whether the cardiac phenotype in the cohort of patients is also detected in canonical Leigh syndrome should be specified.

Genotype–phenotype correlations could be better discussed. For example, it would be informative to highlight whether the extent of residual COX activity or COXFA4L2 expression correlates with clinical severity or age of onset. Patient S3, who displays comparatively high COXFA4L2 expression and a milder disease course, is particularly noteworthy. A more explicit discussion of this potential association would help establish the clinical relevance of the proposed compensatory mechanism.

The demonstration that COXFA4L2 is transcriptionally upregulated in all patient fibroblasts represents one of the key findings of the manuscript. The quantitative PCR data convincingly show the absence of COXFA4 transcripts and compensatory induction of COXFA4L2. The immunoblots are interpreted as confirmation that COXFA4L2 protein levels increase in the patient lines, but the data are difficult to interpret because the antibodies used do not discriminate perfectly between the two paralogs. Thus, it would be helpful to improve the quality of the blots.

The observation that COXFA4L2 upregulation is particularly pronounced in S3 fibroblasts would deserve better discussion. The authors might consider whether this correlates with the relatively mild clinical phenotype in that individual. Such an analysis could provide an elegant link between molecular compensation and disease outcome.

The authors exclude a general response to COX dysfunction by examining SURF1-mutant fibroblasts, yet they do not investigate whether inflammatory or stress-related pathways contribute to COXFA4L2 induction. Given that COXFA4L3 is strongly induced by inflammatory cues (Clayton et al Science Advances, 2021), this possibility should at least be discussed, even if direct evidence is lacking.

The BNGE data in Figure 3 show a clear reduction in assembled complex IV in all COXFA4-deficient fibroblasts. This is consistent with the reported loss of COXFA4 protein. However, the accompanying claim that complex I and III_2 are unaffected seems overstated. The gel shown in Figure 3 suggests that complex I abundance might actually be increased in several samples. The authors should reconsider this point. Also, it is unclear why the authors use in-gel activity assays rather than immunoblotting to assess complex I in Figure 4 and Supplemental Figure 7C. The IGA sensitivity may be lower than that of a an antibody, potentially masking real variation. The use of a complex I-specific antibody would clarify this point.

The finding of increased complex V assembly is intriguing and might indicate a compensatory attempt to preserve ATP production under impaired COX function. This could be elaborated upon in the discussion. The authors should also downsize the statement that that COXFA4 is directly required for “supercomplex integration.” The data are more consistent with a primary defect in COX holoenzyme assembly, with secondary consequences for the formation of respirasomes. The increase in free complex III_2 supports this interpretation.

The pulse–chase experiments using doxycycline to inhibit mitochondrial translation aim to demonstrate delayed assembly kinetics of COX holoenzyme and supercomplexes in the absence of COXFA4. While conceptually sound, this section is difficult to follow. The description of timing, normalization, and relative band intensities should be better explained. I also suggest including some kinetic parameters to amke this section more quantitative. Moreover, COXFA4L2 is stated to be “undetectable” in these assays, yet this contradicts earlier data showing its strong upregulation in the same fibroblast line (S1). Clarification is needed to reconcile these findings, which might relate to antibody specificity rather than true absence of COXFA4L2.

The complementation experiments elegantly demonstrate that re-expression of wild-type COXFA4 restores complex IV activity in patient fibroblasts, confirming the pathogenicity of the variants. Expression of COXFA4L2 also enhances COX activity, albeit less effectively, providing direct evidence for partial functional redundancy.
The knockdown experiments, however, raise interpretative questions. The authors report that depletion of COXFA4L2 in COXFA4-deficient fibroblasts reduces maximal respiration while leaving basal respiration unchanged, implying that COXFA4L2 contributes primarily to spare respiratory capacity. This is an important observation, but the term “spare capacity” should be briefly defined for clarity, and the potential cellular consequences of this reduction should be elaborated upon. The discussion could also explore more concretely how COXFA4L2 expression might be therapeutically modulated. While

the authors mention potential translational relevance, they do not suggest plausible strategies.

The SH-SY5Y knockdown model nicely extends the findings to a neuronal context. The authors show that loss of COXFA4 in these cells induces COXFA4L2 expression, supporting a conserved compensatory mechanism. However, the rationale for including additional cell lines (H4, A549, HeLa, HEK293) is not well explained, and the apparent increase of COXFA4L2 in SH-SY5Y cells is modest and not obvious from the blot. Clarifying which of these lines are neuroblastoma versus non-neuronal would aid interpretation. The reference to H4 neuroglioma cells in this section is confusing and should be streamlined for consistency.
The discussion effectively situates this work within the context of mitochondrial disease genetics and complex IV biology. Nevertheless, while the authors propose COXFA4L2 modulation as a potential therapeutic avenue, the statement remains vague. Suggesting specific experimental or pharmacological approaches would make this more credible and could stimulate follow-up work.

Reviewer #3

(Remarks to the Author)
The paper by Falabella et al. describes the clinical presentation of 13 individuals from 12 unrelated families with an ultra-rare mitochondrial disease caused by biallelic pathogenic variants in the COXFA4 gene. The defect in COXFA4 results in defective supercomplex formation and isolated COX defect. Transcriptomic analysis revealed upregulation of COXFA4L2, a poorly characterized gene which has otherwise limited expression in most tissues. The authors demonstrated that COXFA4L2 partially compensates for the loss of COXFA4.

It is a very inteersting paper and provides important novel insights about the assembly and function of COX and it effect on other mitochondrial respiratory chain enzymes.

1.I think the detailed clinical description of the families can go to the supplement. Instead it would be good to have a summarizing text about the overall phenotypic characteristics of the patients.
2. Are there any genotype-phenotype correlations? The authors show that there is a low amount of normal splicing possible in the splice-site mutations, which may result in a low amount of residual protein (possibly not detectable on western blot). Is the phenotype of patients milder with these splice site mutations?
3. The description of the splicing defects of the various mutations is quite complicated. It should be better explained.
4. If COXFA4 defect impairs supercomplex formation, why are the other complexes in the supercomplex unaffected? Please, provide some hypothesis.
5. Why did the authors select SH-SY5Y cells to study whether double KD of both COXFA4 and COXFA4L2 is more severely affected, if COXFA4L2 is not known to be expressed in astrocytes or neurons?
6. The double KD in SH-SY5Y cells resulted in more reduced basal respiration on Seahorse analysis than KD of COXFA4 only. Has it also led to more severe reduction of COX subunits or COX activity?
6. The brain MRI images are quite interesting. I suggest adding this to the main Figure 1 if possible.

Version 1:

Reviewer comments:

Reviewer #1

(Remarks to the Author)
The authors have responded to all my comments satisfactorily.

Reviewer #2

(Remarks to the Author)
I appreciate the effort the authors made to address my concerns and comments. This definitely strengthened the manuscript. I have no further comments.

Reviewer #3

(Remarks to the Author)
The authors addressed all my questions and imporved the paper. I do not have any more comments.

Open Access This Peer Review File is licensed under a Creative Commons Attribution 4.0 International License, which permits use, sharing, adaptation, distribution and reproduction in any medium or format, as long as you give appropriate credit to the original author(s) and the source, provide a link to the Creative Commons license, and indicate if changes were made.
In cases where reviewers are anonymous, credit should be given to 'Anonymous Referee' and the source.
The images or other third party material in this Peer Review File are included in the article's Creative Commons license, unless indicated otherwise in a credit line to the material. If material is not included in the article's Creative Commons license and your intended use is not permitted by statutory regulation or exceeds the permitted use, you will need to obtain permission directly from the copyright holder.
To view a copy of this license, visit https://creativecommons.org/licenses/by/4.0/

Response to Reviewers

We thank the Reviewers for their constructive and insightful comments. We have revised the manuscript accordingly. Below, we provide a point-by-point response to each comment. All changes in the manuscript are highlighted in yellow.

Reviewer 1: Comments to Author

This manuscript reports a large cohort of patients with a Leigh-like phenotype (neurodegenerative disorder) all of whom have biallelic pathogenic variants in COXFA4, a subunit of Complex IV (cytochrome c oxidase). The mutations in the gene are all loss of function, and the protein is undetectable by immunoblot analysis, suggesting that all patients are functionally null for the protein. The loss of COXFA4 results in the upregulation of a paralogue COXFA4L2, which partially compensates for the loss of complex IV activity analyzed in patient fibroblasts. The genetic analysis is completely convincing, and the authors do a rather thorough job of downstream biochemical analysis. This represents an important addition to our understanding of primary mitochondrial disease, and in particular those associated with impaired complex IV activity

I have a few comments/queries that I think should be addressed to improve the manuscript.

1. *In some experiments primary patient fibroblasts are used in others immortalized fibroblasts. There are no descriptions of culture conditions or the agent(s) used to immortalize the cells. Do the immortalized cells remain diploid?*

Primary and immortalised fibroblasts were cultured under identical conditions, using high-glucose Dulbecco's Modified Eagle Medium supplemented with 4 mM glutamine, 110 mg/L pyruvate, 10% (v/v) fetal bovine serum (Gibco, Life Technologies), 100 U/ml penicillin/streptomycin (Gibco, Life Technologies). Fibroblasts were immortalised using the pLOX-Ttag-iresTK vector, which expresses the SV40 large T antigen. These details are now included in the Supplementary Information. We did not perform a

karyotype analysis on the immortalised lines, and we cannot exclude the possibility of chromosomal alterations. However, the immortalised cells retained fibroblast morphology and key molecular features of primary cells. The immortalised lines were used in Figure 4 (now Figure 5) and Supplementary Figures 7 and 8 (now Supplementary Figures 6 and 7). Importantly, these findings are consistent with those observed in the corresponding primary fibroblast lines, indicating that the reported effects are not driven by the immortalisation process.

2. *There seems to be a lot of confusion about the specificity of the antibodies used to detect COXFA4 and its paralogue. In the methods section it is stated that the anti-COXFA4 recognizes both proteins. Yet on pg 22 I read the following "This is consistent with the high specificity of the COXFA4 antibody, which does not react with its paralog". Clearly both statements cannot be true, and the data in Fig. 2D show that the antibody detects both. Further, in Figs. 2E,F it is stated that an antibody specific for COXFA4L2 was used but the data shown suggest otherwise. Looks to me that it recognizes both proteins.*

We thank the Reviewer for highlighting this inconsistency. In this study, we used two different antibodies targeting COXFA4: Stratech Scientific (C16821-ABT) and Abcam (ab129752). The Stratech Scientific antibody (C16821-ABT) recognises both COXFA4 and its paralogue COXFA4L2 and was used in all experiments except those shown in Figure 4 (now Figure 5) and Supplementary Figures 7 and 8 (now Supplementary Figures 6 and 7). The Abcam antibody, used in Figure 4 (now Figure 5) and Supplementary Figures 7 and 8 (now Supplementary Figures 6 and 7), preferentially detects COXFA4 and does not detect COXFA4L2 at low basal expression levels. We also agree with the Reviewer that the antibody used to detect COXFA4L2 (Proteintech, 16480-1-AP) recognises both COXFA4 and COXFA4L2. We have corrected the statement on page 22 and clarified this point throughout the manuscript. The antibody used in each experiment is now explicitly specified in the corresponding figure legends.

3. *I am also puzzled why the antibody directed against the paralog was used in Fig 2.G. COXFA4L2 is barely detectable in control cells, and that is not consistent with detection of the native complex at relatively high levels. Seems to me that they should have used an antibody that is known to recognize complex IV on native gels, otherwise I think the results in this figure are not readily interpretable. Why for instance is the amount of the complex similar in controls and patients. There should be considerably of the fully assembled complex in the patient. I would also like to be convinced that the antibody can recognize the native complex.*

In Figure 2G (now Figure 3G), we used the Proteintech antibody (16480-1-AP), which is reported to recognise COXFA4L2, but we show that it also detects COXFA4 (now Figure 3E and 3F). This cross-reactivity made the antibody suitable for monitoring the association of both paralogues with native complex IV on BN-PAGE. The aim of this experiment was to assess the stability of COXFA4- or COXFA4L2-containing complexes under increasing detergent concentrations. Previous studies have shown that COXFA4 is weakly associated with complex IV and dissociates at DDM concentrations higher than 0.02% (Pitceathly et al., 2013). We therefore performed a DDM titration to test whether COXFA4L2 exhibits a similar association pattern in patient cells. The persistence of the signal at higher DDM concentrations indicates a more stable association of COXFA4L2 with the complex IV, rather than a similar overall amount of fully assembled complex IV between controls and patients. Quantitative changes in complex IV abundance and assembly were analysed separately using antibodies against core complex IV subunits (now Figures 4 and 5; Supplementary Figures 6 and 7). We agree that antibody choice is critical for BN-PAGE interpretation, and we have clarified in the Results and the Figure 2G (now Figure 3G) legend that this experiment specifically assesses the detergent stability of the COXFA4/COXFA4L2 association with complex IV, rather than overall complex IV abundance.

4. *I think it is difficult to conclude that the lack of COXFA4 in and of itself impedes respirasome assembly. If there is less of the "free" complex IV, seems to me there will be less in the respirasome. That doesn't necessarily imply that the subunit is somehow necessary for higher order complexes.*

We have revised the manuscript to avoid implying a direct requirement of COXFA4 for supercomplex assembly. We now describe supercomplex alterations as secondary consequences of impaired COX holoenzyme assembly.

5. *Also, in Fig 4. what accounts for the very low levels of complex II? It does not have mtDNA-encoded subunits so should not be affected by doxycycline.*

Complex II is entirely nuclear-encoded and is therefore not expected to be directly affected by doxycycline-mediated inhibition of mitochondrial translation. Consistent with this, SDHA steady-state levels assessed by SDS–PAGE remain stable across time points (now Supplementary Figure 7). For this reason, SDHA was used as a reference for normalisation in these experiments. The apparent changes in SDHA signal intensity across time points in Figure 4 (now Figure 5) reflect differences in gel loading, rather than a biological reduction in complex II abundance.

6. *Further, the data presented in Fig. 4 seem at odds with the data presented in Supp. Fig 8, and I wonder why the antibody against the paralog was not used in the latter experiment.*

The same antibody (Abcam ab129752) was used in both Figure 4 (now Figure 5) and Supplementary Figure 8 (now Supplementary Figure 7), and we have now added this information to the figure legend. This antibody preferentially detects COXFA4 and does not reliably detect COXFA4L2 at its low basal expression levels. Figure 4 (now Figure 5) shows the reassembly of fully assembled complex IV and supercomplexes by BN-PAGE. At early time points following doxycycline withdrawal, fully assembled complex IV has not yet formed, which explains the weak or absent signal. In contrast, Supplementary Figure 8 (now Supplementary Figure 7) analyses the reappearance of individual mitochondrial-encoded COX subunits by SDS–PAGE, which precedes the formation of the fully assembled holoenzyme. This explains the apparent discrepancy

between the two figures as they capture different stages of the biogenesis process. We did not use the anti-COXFA4L2 antibody in these experiments because the purpose of these assays was to track canonical COX assembly kinetics. We have clarified the distinction between the two assays in the manuscript.

7. *Maybe I am missing something, but I don't understand why low concentrations of DDM do not solubilize the complex from the patient.*

As previously reported, COXFA4 is weakly associated with the COX holoenzyme and dissociates at detergent concentrations commonly used for BN-PAGE (>0.02%) (Pitceathly et al., 2013). We therefore performed a DDM titration at lower concentrations to compare the stability of COX association with COXFA4 (in controls) versus COXFA4L2 (in patients). Our *in silico* modelling predicts a higher binding affinity of COXFA4L2 for the COX complex, providing a mechanistic explanation for why the complex remains detectable at higher DDM concentrations in patient cells. Importantly, the increased signal at higher DDM concentrations reflects greater subunit stability, not increased complex IV abundance.

8. *I do not get Supp Fig 6 C. On the blot is says the COXFA4L2 antibody was used. In the legend it says the COXFA4 antibody was used. Further, these results do not accord with those in Fig 4 where the complex was seen at low DDM concentrations in controls but not in the patient. Explanation?*

We thank the Reviewer for highlighting this point. We have amended the legend of Supplementary Figure 6C (now Supplementary Figure 5C) to state that the anti-COXFA4L2 antibody (Proteintech, 16480-1-AP) was used.

Regarding the apparent discrepancy in Figure 4 (now Figure 5), these two experiments assess distinct aspects of complex IV biology. Supplementary Figure 6C (now Supplementary Figure 5C) tests the detergent-dependent stability of pre-existing COX complexes using n-dodecyl β-D-maltoside (DDM), whereas Figure 4 (now Figure 5), shows the kinetics of *de novo* complex and supercomplex assembly after reversible inhibition of mitochondrial translation by doxycycline. Thus, Supplementary Figure 6C (now Supplementary Figure 5C) reports on the stability of already assembled

complexes, while Figure 4 (now Figure 5) tracks the temporal formation of new complexes.

9. *I do not think it is possible to conclude anything definitive about the order of assembly of the subunits in complex IV from the data presented in this manuscript.*

We agree with the Reviewer that our data do not allow us to define the precise temporal order of incorporation of individual subunits into complex IV. Our results are consistent with a role for COXFA4 at a late stage of COX biogenesis. This is based on delayed holoenzyme assembly and preserved formation of earlier intermediates. We have revised the manuscript to avoid definitive claims on the order of subunit incorporation.

10. *Very minor - I would settle on immunoblot or Western blot. Personally, I prefer the former. Changed to immunoblot.*

Changed to immunoblot

Reviewer 2: Comments to Author

This manuscript by Falabella and colleagues presents an extensive investigation that significantly advances our understanding of COXFA4-related mitochondrial disease. The authors describe 13 affected individuals from 12 unrelated families carrying biallelic pathogenic variants in COXFA4. Using a combination of genomic, transcriptomic, biochemical, and structural approaches, they demonstrate that loss of COXFA4 protein leads to a marked reduction in complex IV activity, yet residual enzymatic function is preserved through upregulation of COXFA4L2, a paralog with minimal basal expression. Functional studies in patient-derived fibroblasts and neuronal cells, combined with complementation and knockdown assays, support a model in which COXFA4L2 partially substitutes for COXFA4, maintaining limited COX activity and respiratory capacity.

The study is interesting, well written and the experiments elegant. This work will thus likely have considerable impact in the mitochondrial disease field. However, several aspects require clarification.

1. *The genetic and clinical characterization of the cohort is extremely accurate and consistent with a Leigh-like phenotype. The identification of seven novel variants, including multiple splice-site mutations, adds valuable information to the existing COXFA4 mutational spectrum. However, whether the cardiac phenotype in the cohort of patients is also detected in canonical Leigh syndrome should be specified.*

Done.

2. *Genotype–phenotype correlations could be better discussed. For example, it would be informative to highlight whether the extent of residual COX activity or COXFA4L2 expression correlates with clinical severity or age of onset. Patient S3, who displays comparatively high COXFA4L2 expression and a milder disease course, is particularly noteworthy. A more explicit discussion of this potential association would help establish the clinical relevance of the proposed compensatory mechanism.*

We agree with the Reviewer that Patient S3 is particularly interesting, as this individual showed higher COXFA4L2 mRNA and protein levels together with greater residual COX activity and a milder clinical course. We have now included this point in the Discussion. However, we also emphasise that establishing a causal relationship remains challenging at this stage. Importantly, the two other individuals with the same variant (S1 and S2) did not show a comparable increase in COXFA4L2 expression in fibroblasts, indicating that this response is not uniform across lines. Moreover, since fibroblasts may not fully recapitulate disease-relevant tissues such as the brain, it remains unclear how COXFA4L2 upregulation is regulated in target tissues. Additional studies in disease-relevant cell types and larger cohorts will be required to clarify the mechanisms underlying COXFA4L2 induction and its contribution to clinical phenotypes.

3. *The demonstration that COXFA4L2 is transcriptionally upregulated in all patient fibroblasts represents one of the key findings of the manuscript. The quantitative PCR data convincingly show the absence of COXFA4 transcripts and compensatory induction of COXFA4L2. The immunoblots are interpreted as confirmation that COXFA4L2 protein levels increase in the patient lines, but the data are difficult to interpret because the antibodies used do not discriminate perfectly between the two paralogs. Thus, it would be helpful to improve the quality of the blots.*

We agree with the Reviewer that discrimination between COXFA4 and COXFA4L2 at the protein level is technically challenging due to their similar molecular weights (9.4 vs 10 kDa) and the lack of COXFA4L2-specific antibodies. We attempted to further optimise these immunoblots during revision; however, this was limited by the senescence of several patient-derived fibroblast lines and the difficulty of resolving very low–molecular–weight proteins. Importantly, siRNA-mediated knockdown of COXFA4L2 selectively reduced the upper band (Figure 3F and Supplementary Figure 5A), while COXFA4L2 overexpression specifically increased this same band (Figure 6C). Together, these experiments confirm that the upper band corresponds to COXFA4L2 and demonstrate that COXFA4L2 is upregulated at both the transcript and protein levels in patient cells.

4. *The observation that COXFA4L2 upregulation is particularly pronounced in S3 fibroblasts would deserve better discussion. The authors might consider whether this correlates with the relatively mild clinical phenotype in that individual.*

Done.

5. *The authors exclude a general response to COX dysfunction by examining SURF1-mutant fibroblasts, yet they do not investigate whether inflammatory or stress-related pathways contribute to COXFA4L2 induction. Given that COXFA4L3 is strongly induced by inflammatory cues (Clayton et al Science Advances, 2021), this possibility should at least be discussed, even if direct evidence is lacking.*

Done.

6. *The BNGE data in Figure 3 show a clear reduction in assembled complex IV in all COXFA4-deficient fibroblasts. This is consistent with the reported loss of COXFA4 protein. However, the accompanying claim that complex I and III_2 are unaffected seems overstated. The gel shown in Figure 3 suggests that complex I abundance might actually be increased in several samples. The authors should reconsider this point.*

Done.

7. *Also, it is unclear why the authors use in-gel activity assays rather than immunoblotting to assess complex I in Figure 4 and Supplemental Figure 7C. The IGA sensitivity may be lower than that of an antibody, potentially masking real variation. The use of a complex I-specific antibody would clarify this point.*

We agree with the Reviewer that immunoblotting with a complex I-specific antibody would provide higher sensitivity than in-gel activity (IGA) assays. Importantly, in the cell line (S1) used for the kinetic experiments in Figure 4 (now Figure 5), we did not observe any defect in complex I assembly or abundance (Figure 3A, now Figure 4A), and therefore, we did not expect major changes during the recovery time course.

8. *The finding of increased complex V assembly is intriguing and might indicate a compensatory attempt to preserve ATP production under impaired COX function. This could be elaborated upon in the discussion.*

Done.

9. *The authors should also downsize the statement that that COXFA4 is directly required for "supercomplex integration." The data are more consistent with a primary defect in COX holoenzyme assembly, with secondary consequences for the formation of respirasomes. The increase in free complex III_2 supports this interpretation.*

We have revised the manuscript to reflect that COXFA4 is not directly required for supercomplex assembly and that the alterations observed are secondary to defects in COX holoenzyme assembly.

10. *The pulse–chase experiments using doxycycline to inhibit mitochondrial translation aim to demonstrate delayed assembly kinetics of COX holoenzyme and supercomplexes in the absence of COXFA4. While conceptually sound, this section is difficult to follow. The description of timing, normalization, and relative band intensities should be better explained. I also suggest including some kinetic parameters to make this section more quantitative.*

The suggested changes have been incorporated into the Methods, Results, and Figure 5 legend.

11. *Moreover, COXFA4L2 is stated to be "undetectable" in these assays, yet this contradicts earlier data showing its strong upregulation in the same fibroblast line (S1). Clarification is needed to reconcile these findings, which might relate to antibody specificity rather than true absence of COXFA4L2.*

This apparent discrepancy is due to the use of different antibodies. The Stratech Scientific antibody (C16821-ABT), which recognises both COXFA4 and COXFA4L2, was used in all experiments except those shown in Figure 5 and Supplementary Figures 6 and 7, where the Abcam antibody (ab129752) was used. In our experimental conditions, the Abcam antibody (ab129752) does not COXFA4L2 at low expression levels. Therefore, we agree with the Reviewer that the apparent "absence" of COXFA4L2 in these assays reflects antibody sensitivity rather than the true absence

of the protein. We have now clarified this throughout the manuscript and specified the antibody used in each experiment.

12 *The complementation experiments elegantly demonstrate that re-expression of wild-type COXFA4 restores complex IV activity in patient fibroblasts, confirming the pathogenicity of the variants. Expression of COXFA4L2 also enhances COX activity, albeit less effectively, providing direct evidence for partial functional redundancy. The knockdown experiments, however, raise interpretative questions. The authors report that depletion of COXFA4L2 in COXFA4-deficient fibroblasts reduces maximal respiration while leaving basal respiration unchanged, implying that COXFA4L2 contributes primarily to spare respiratory capacity. This is an important observation, but the term "spare capacity" should be briefly defined for clarity, and the potential cellular consequences of this reduction should be elaborated upon.*

Done.

13 *The discussion could also explore more concretely how COXFA4L2 expression might be therapeutically modulated. While the authors mention potential translational relevance, they do not suggest plausible strategies.*

Done.

14 *The SH-SY5Y knockdown model nicely extends the findings to a neuronal context. The authors show that loss of COXFA4 in these cells induces COXFA4L2 expression, supporting a conserved compensatory mechanism. However, the rationale for including additional cell lines (H4, A549, HeLa, HEK293) is not well explained, and the apparent increase of COXFA4L2 in SH-SY5Y cells is modest and not obvious from the blot. Clarifying which of these lines are neuroblastoma versus non-neuronal would aid interpretation.*

The additional cell lines (H4, A549, HeLa, HEK293) were included in an initial screen to determine whether basal COXFA4L2 expression is detectable across neuronal- and

non-neuronal lines. We now clarify in the manuscript that SH-SY5Y is a neuroblastoma line, H4 is a neuroglioma line, and the remaining lines are non-neuronal models.

15 *The reference to H4 neuroglioma cells in this section is confusing and should be streamlined for consistency.*

Done.

16 *The discussion effectively situates this work within the context of mitochondrial disease genetics and complex IV biology. Nevertheless, while the authors propose COXFA4L2 modulation as a potential therapeutic avenue, the statement remains vague. Suggesting specific experimental or pharmacological approaches would make this more credible and could stimulate follow-up work.*

Done.

Reviewer 3: Comments to Author

The paper by Falabella et al. describes the clinical presentation of 13 individuals from 12 unrelated families with an ultra-rare mitochondrial disease caused by biallelic pathogenic variants in the COXFA4 gene. The defect in COXFA4 results in defective supercomplex formation and isolated COX defect. Transcriptomic analysis revealed upregulation of COXFA4L2, a poorly characterized gene which has otherwise limited expression in most tissues. The authors demonstrated that COXFA4L2 partially compensates for the loss of COXFA4.

It is a very interesting paper and provides important novel insights about the assembly and function of COX and its effect on other mitochondrial respiratory chain enzymes.

1. *I think the detailed clinical description of the families can go to the supplement. Instead, it would be good to have a summarising text about the overall phenotypic characteristics of the patients.*

We have included a summarising text in the Results section. The detailed individual case descriptions are now provided in the Supplementary Information.

2. *Are there any genotype-phenotype correlations? The authors show that there is a low amount of normal splicing possible in the splice-site mutations, which may result in a low amount of residual protein (possibly not detectable on western blot). Is the phenotype of patients milder with these splice site mutations?*

We have revised the Discussion to provide a potential explanation of the genotype-phenotype relationships, focusing on the modifying role of COXFA4L2 expression and residual COX activity. Although we detected low-level transcript leakage (13.8% and 3.6%) in two cases, it did not consistently correlate with clinical severity. Our data suggest that paralog compensation is more likely to affect remaining COX activity and differences in phenotype. However, how this compensatory response is regulated in disease-relevant tissues remains an open question that may differ between individuals.

3. *The description of the splicing defects of the various mutations is quite complicated. It should be better explained.*

Done.

4. *If COXFA4 defect impairs supercomplex formation, why are the other complexes in the supercomplex unaffected? Please, provide some hypothesis.*

We have revised the manuscript to downplay the direct role of COXFA4 in supercomplex assembly. Our data support a primary defect in COX holoenzyme assembly/stability, with secondary effects on respirasome formation due to reduced availability of fully assembled COX. Impaired COX incorporation leads to fewer complete supercomplexes and accumulation of free complex III_2.

5. *Why did the authors select SH-SY5Y cells to study whether double KD of both COXFA4 and COXFA4L2 is more severely affected, if COXFA4L2 is not known to be expressed in astrocytes or neurons?*

We selected the SH-SY5Y cell line as it is commonly used as a neuronal-like model. While COXFA4L2 is not generally reported to be expressed in primary neurons *in vivo*,

we detected low basal levels in SH-SY5Y, which increased following COXFA4 knockdown. This provided a useful system to test the compensatory potential of COXFA4L2. We now clarify this rationale and the limitations of immortalised cell models in the manuscript.

6. *The double KD in SH-SY5Y cells resulted in more reduced basal respiration on Seahorse analysis than KD of COXFA4 only. Has it also led to more severe reduction of COX subunits or COX activity?*

Our initial aim was indeed to determine whether the more pronounced reduction in basal respiration observed in the double knockdown was also reflected at the level of COX activity and COX subunit abundance. To this end, we attempted to perform both spectrophotometric COX activity assays in the double knockdown SH-SY5Y cells. However, the low viability of the double knockdown line prevented the collection of sufficient material for mitochondrial isolation and biochemical analyses.

7. *The brain MRI images are quite interesting. I suggest adding this to the main Figure 1 if possible.*

We thank the Reviewer for this suggestion and agree that the brain MRI images are highly informative. However, Figure 1 is already very dense, and adding the MRI panels would require substantial downscaling, reducing image quality. We have therefore placed them in the main Figure 2, where they can be shown at sufficient size and resolution.
